# Polyoxygenated Sterols from the South China Sea Soft Coral *Sinularia* sp

**DOI:** 10.3390/md10071422

**Published:** 2012-06-26

**Authors:** Rui Li, Chang-Lun Shao, Xin Qi, Xiu-Bao Li, Jing Li, Ling-Ling Sun, Chang-Yun Wang

**Affiliations:** 1 Key Laboratory of Marine Drugs, Ministry of Education of China, School of Medicine and Pharmacy, Ocean University of China, Qingdao 266003, China; Email: lirui30502@163.com (R.L.); shaochanglun@163.com (C.-L.S.); qxhin@163.com (X.Q.); ljlilac@163.com (J.L.); sunllqd@163.com (L.-L.S.); 2 South China Sea Institute of Oceanology, Chinese Academy of Sciences, Guangzhou 510000, China; Email: lixiubao@scsio.ac.cn

**Keywords:** soft coral, *Sinularia* sp., polyoxygenated sterols, cytotoxicity

## Abstract

Chemical investigation of the ethanol extract of soft coral *Sinularia* sp. collected from the South China Sea led to the isolation of three new polyoxygenated sterols, (3*S*,23*R*,24*S*)-ergost-5-ene-3β,23α,25-triol (**1**), (24*S*)-ergostane-6-acetate-3β,5α,6β,25-tetraol (**2**), (24*S*)-ergostane-6-acetate-3β,6β,12β,25-tetraol (**3**) together with three known ones (**4**–**6**). The structures, including relative configurations of the new compounds (**1**–**3**), were elucidated by detailed analysis of spectroscopic data (IR, UV, NMR, MS) and by comparison with related reported compounds. The absolute configuration of **1** was further determined by modified Mosher’s method. Compound **5** exhibited moderate cytotoxicity against K562 cell line with an IC_50_ value of 3.18 μM, but also displayed strong lethality toward the brine shrimp *Artemia salina* with a LC_50_ value of 0.96 μM.

## 1. Introduction

**Figure 1:**
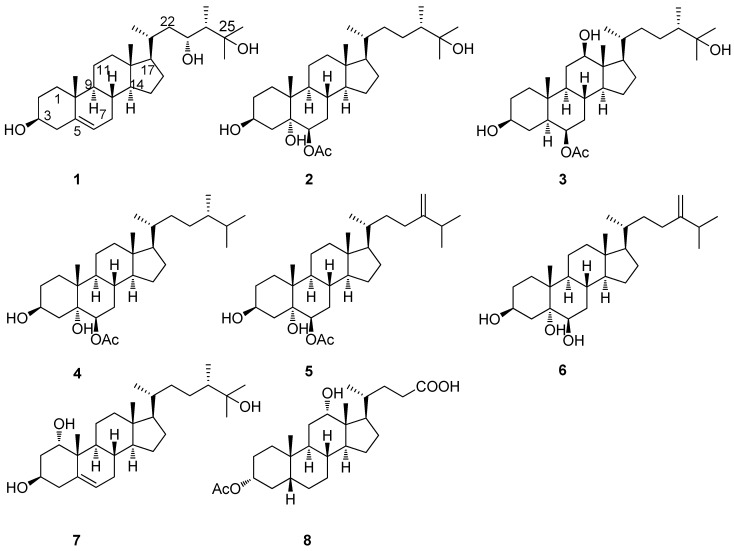
Structures of compounds **1**–**6** from *Sinularia* sp.

Soft coral of the genus *Sinularia* has been found to be a rich source of bioactive secondary metabolites [1,2], such as acylated spermidine [3,4], lipids and fatty acids [5], cyclic sesquiterpene peroxides [6], sterols [7,8], and norditerpenes [9,10]. A number of them showed an array of biological activities such as cytotoxic activities [3,4] and inhibitory effect on LPS-induced TNF-α production [9,10]. As part of our ongoing investigation of new natural bioactive compounds from marine invertebrates in the South China Sea [11,12,13,14,15,16], the soft coral *Sinularia* sp. attracted our attention because the crude extract of *Sinularia* sp. showed lethal activity toward brine shrimp *Artemia salina*. Bioassay-guided fractionation of the active extracts led to the isolation of three new polyoxygenated sterols (**1**–**3**) and three known ones (**4**–**6**) [17,18] (Figure 1).

## 2. Results and Discussion

**Figure 2:**
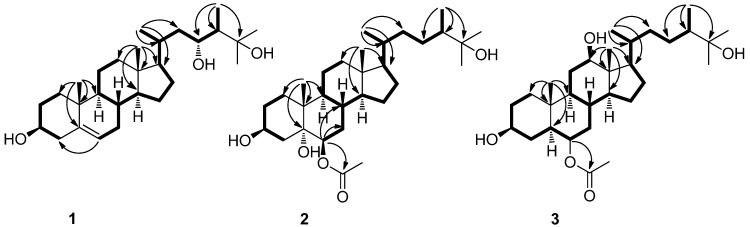
^1^H-^1^H COSY(▬) and HMBC (→) correlations for compounds **1**–**3**.

**Figure 3:**
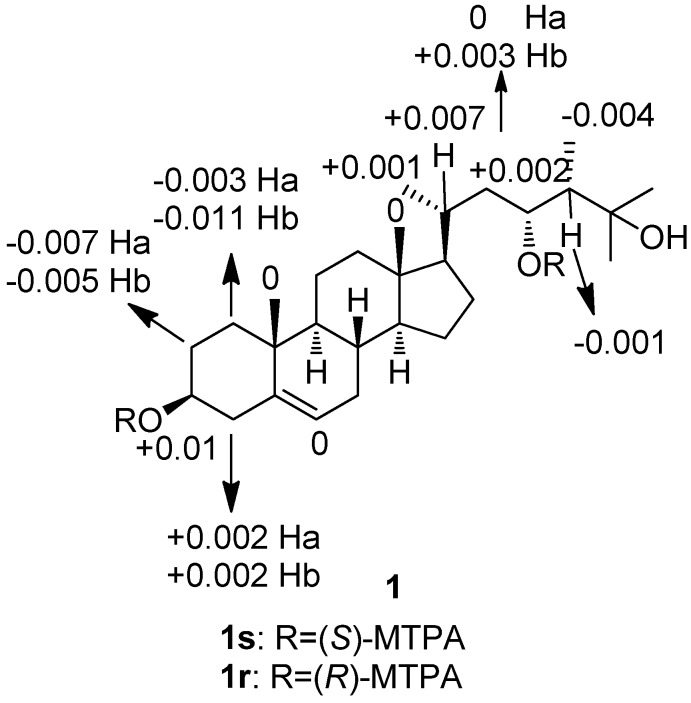
Δ*δ* values (*δ*_(*S*)_ − *δ*_(*R*)_) for the MTPA esters of compound **1**.

Compound **1** was obtained as a white, amorphous powder with [α]_D_^25^ −22.4 (*c* 0.05, CH_3_OH). IR spectrum showed its absorption at 3410–3584 cm^−1^ for hydroxy groups. The molecular formula was established as C_28_H_48_O_3_ based on HRESIMS ([M + Na]^+^ 455.3498 (calcd for C_28_H_48_O_3_Na 455.3496)) and NMR spectroscopic data. The ^1^H-NMR (Table 1) and ^13^C-NMR (Table 2) spectra implied that compound **1** was a polyhydroxylated sterol. The ^1^H-NMR spectrum of **1** revealed four methyl singlet signals at *δ*_H_ 0.70 (3H, s, H_3_-18), 1.00 (3H, s, H_3_-19), 1.23 (3H, s, H_3_-26) and 1.23 (3H, s, H_3_-27), and two methyl doublet signals at *δ*_H_ 0.81 (3H, d, *J* = 6.6 Hz, H_3_-28) and 1.08 (3H, d, *J* = 6.6 Hz, H_3_-21), as well as two oxymethines (*δ*_H_ 3.71 and 3.56), and an olefinic proton signal at *δ*_H_ 5.34 (br d, *J* = 4.8 Hz). The ^13^C-NMR and DEPT spectra of **1** displayed 28 signals which were assigned to four quaternary carbons, nine methines, nine methylenes, and six methyls. The olefinic carbon signals appearing at *δ*_C_ 140.8 (C) and 121.6 (CH) corresponded to one trisubstituted double bond. The ^13^C-NMR chemical shifts at *δ*_C_ 75.8 (CH), 75.2 (C) and 71.8 (CH) confirmed the presence of three oxygenated carbons. Detailed analysis of the ^1^H-^1^H COSY spectrum in combination with HMQC and HMBC (Figure 2) experiments allowed the assignment of all of the chemical shifts in the ^1^H and ^13^C-NMR spectra and led to structure **1**. Comparison of ^1^H-NMR and ^13^C-NMR of **1** with those of the known compound **7** (patusterol A) [19], a hydroxylated steroid from the Kenyan soft coral *Lobophytum patulum*, further confirmed the structure of **1**. The obvious differences between the two compounds are the chemical shifts at *δ*_H_3.71 (1H, ddd, *J* = 12.0, 8.4, 3.0 Hz, H-23) in **1** *vs.* 3.71 (1H, br t,*J* = 2.6 Hz, H-1) in **7**, and *δ*_C_ 75.8 (CH) in **1** *vs.* 72.5 (CH) in **7**, indicating that the hydroxyl group in **1** is at C-23 not as **7** at C-1. In addition, the ^1^H–^1^H COSY correlations of H-23 with H-24 and H-22, and the HMBC correlations from H_3_-28 to C-24, C-23 and C-25 enable the hydroxyl group to be placed at C-23. The relative stereochemistry of **1 **was assigned on the basis of 2D NOESY experiment. In the NOE spectrum of **1**, NOE correlations observed from H_3_-18 to H-20 indicated that H-20 was in β disposition. The α orientation of H-9 and H-14, and β orientation of H-8 were also determined by NOESY experiment. The absolute configuration at C-3 and C-23 of **1** was tried to determine using the modified Mosher’s method [20]. The (*S*)- and (*R*)-MTPA esters **1r** and **1s **were prepared using (*R*)- and (*S*)-MTPA chloride, respectively. The determination of Δ*δ* values (*δ_S_* − *δ_R_*) (Figure 3) for protons neighboring C-3 and C-23 should lead to the assignment of the configuration at C-3 and C-23 in **1**. The configuration at C-24 in this and congener sterols was suggested as 24*S* on biogenetic grounds, since almost all the 24-methylsterols isolated from corals have 24*S* stereochemistry according to the literature [21,22]. According to the relatively small difference of Δ*δ* values (*δ_S_* − *δ_R_*), the absolute configuration of **1** was tentatively determined as (3*S*,23*R*,24*S*)-ergost-5-ene-3β,23α,25-triol.

**Table 1:** ^1^H-NMR data for compounds **1**–**3**.

H#	1, *δ*_H_ (*J* in Hz) *^a^*	2, *δ*_H_ (*J* in Hz) *^b^*	3, *δ*_H_ (*J* in Hz) *^a^*
1	1.82 (1H, d, *J* = 4.8 Hz, H-ax)	1.75 (1H, br d, *J* = 12.0 Hz, H-ax)	1.82 (1H, br d, *J* = 4.8 Hz, H-ax)
	1.12 (1H, m, H-eq)	1.18 (1H, m, H-eq)	1.27 (1H, m, H-eq)
2	2.30 (1H, ddd, *J* = 13.2, 4.8, 1.8 Hz, H-ax)	2.01 (1H, dt, *J* = 12.0, 2.4 Hz, H-ax)	1.99 (1H, 1H, dt, *J* = 12.6, 2.4 Hz, H-ax)
	1.49 (1H, m, H-eq)	1.51 (1H, m, H-eq)	1.49 (1H, m, H-eq)
3	3.56 (1H, m)	3.99 (1H, m)	4.09 (1H, m)
4	2.22 (1H, td, *J* = 12.6, 2.4 Hz, H-ax)	1.77 (1H, br d, *J* = 12.0 Hz, H-ax )	1.84 (1H, br d, *J* = 12.0 Hz, H-ax)
	1.48 (1H, d, *J* = 2.4 Hz, H-eq)	1.53 (1H, m, H-eq)	1.54 (1H, m, H-eq)
5	–	–	1.61 (1H, m) *^d^*
6	5.34 (1H, br d, *J* = 4.8 Hz)	4.68 (1H, t, *J* = 2.4 Hz)	4.70 (1H, m)
7	2.02 (1H, dt, *J* = 12.6, 4.8 Hz, H-ax)	1.68 (1H, dd, *J* =13.8, 2.4 Hz, H-ax)	1.64 (1H, m) **
	1.96 (1H, m, H-eq)	1.47 (1H, d, *J* = 2.4, H-eq)	1.61 (1H, m) *^d^*
8	1.52 (1H, m)	1.52 (1H, m)	1.29 (1H, m)
9	1.16 (1H, m)	1.63 (1H, m)	1.68 (1H, m)
10	–	–	–
11	1.43 (1H, m)	1.34 (1H, m)	1.73 (1H, dd, *J* = 14.4, 7.2 Hz H-ax)
	1.32 (1H, m)	1.32 (1H, m)	0.77 (1H, m, H-eq)
12	1.28 (1H, m)	1.58 (1H, m)	4.31 (1H, td, *J* = 7.2, 0.6 Hz)
	0.97 (1H, m)	1.42 (1H, m)	
13	–	–	–
14	1.33 (1H, m)	1.25 (1H, m)	1.30 (1H, m)
15	1.44 (1H, m)	1.55 (1H, m)	1.51 (1H, m)
	0.94 (1H, m)	1.03 (1H, m)	1.02 (1H, m)
16	1.88 (1H, m)	1.89 (1H, m)	1.86 (1H, m)
	1.45 (1H, m)	1.50 (1H, m)	1.40 (1H, m)
17	1.15 (1H, m)	1.05 (1H, m)	1.10 (1H, m)
18	0.70 (3H, s)	0.78 (3H, s)	0.68 (3H, s)
19	1.00 (3H, s)	1.14 (3H, s)	1.16 (3H, s)
20	1.50 (1H, m)	1.36 (1H, m)	1.39 (1H, m)
21	1.08 (3H, d, *J* = 6.6 Hz)	0.94 (3H, d, *J* = 6.6 Hz)	0.93 (3H, d, *J* = 6.6 Hz)
22	1.84 (1H, m)	1.62 (1H, m)	1.62 (1H, m)
	1.10 (1H, m)	1.01 (1H, m)	1.08 (1H, m)
23	3.71 (1H, ddd, *J* = 12.0, 8.4, 3.0 Hz)	1.68 (1H, m)	1.86 (1H, m)
		0.77 (1H, m)	0.78 (1H, m)
24	1.56 (1H, m)	1.27 (1H, m)	1.28 (1H, m)
25	–	–	–
26	1.23 (3H, s) *^c^*	1.09 (3H, s)	1.15 (3H, s) *^e^*
27	1.23 (3H, s) *^c^*	1.10 (3H, s)	1.15 (3H, s) *^e^*
28	0.81 (3H, d, *J* = 6.6 Hz)	0.87 (3H, d, *J* = 7.2 Hz)	0.89 (3H, d, *J* = 7.2 Hz)
OAc	–	2.02 (3H, s, *CH*_3_CO–)	2.06 (3H, s, *CH*_3_CO–)

*^a^* Spectra were measured in CDCl_3_ (600 MHz); *^b^* Spectra were measured in CD_3_OD (600 MHz). *^c^*^,*d*,*e*^ Overlapping signals.

**Table 2:** ^13^C-NMR data for compounds **1**–**3**.

C#	1, *^a^ δ*_C_, type	2, *^b^ δ*_C_, type	3, *^a^ δ*_C_, type
1	37.2, CH_2_	33.2, CH_2_	34.9, CH_2_
2	24.3, CH_2_	22.2, CH_2_	21.1, CH_2_
3	71.8, CH	67.9, CH	67.3, CH
4	42.3, CH_2_	31.6, CH_2_	28.2, CH_2_
5	140.8, C	75.5, C	30.7, CH
6	121.6, CH	77.8, CH	76.1, CH
7	31.7, CH_2_	32.5, CH_2_	31.4, CH_2_
8	31.9, CH	32.2, CH	31.9, CH
9	50.1, CH	46.2, CH	45.2, CH
10	36.5, C	39.6, C	38.5, C
11	21.1, CH_2_	29.1, CH_2_	40.5, CH_2_
12	39.7, CH_2_	41.0, CH_2_	73.7, CH
13	42.5, C	43.9, C	42.7, C
14	56.6, CH	57.3, CH	55.8, CH
15	21.0, CH_2_	25.2, CH_2_	24.1, CH_2_
16	28.5, CH_2_	29.3, CH_2_	29.7, CH_2_
17	57.3, CH	57.4, CH	55.9, CH
18	11.8, CH_3_	12.6, CH_3_	12.2, CH_3_
19	19.4, CH_3_	17.1, CH_3_	16.5, CH_3_
20	35.0, CH	37.8, CH	36.3, CH
21	23.2, CH_3_	15.3, CH_3_	19.0, CH_3_
22	44.2, CH_2_	36.3, CH_2_	39.9, CH_2_
23	75.8, CH	29.1, CH_2_	30.6, CH_2_
24	48.9, CH	46.4, CH	45.4, CH
25	75.2, C	74.2, C	75.3, C
26	30.7, CH_3_	26.0, CH_3_	26.2, CH_3_
27	30.7, CH_3_	27.2, CH_3_	27.2, CH_3_
28	14.1, CH_3_	19.6, CH_3_	14.8, CH_3_
*CH*_3_CO	–	21.4, CH_3_	21.4, CH_3_
CH_3_ *C*O	–	172.1, C	164.5, C

*^a^* Spectra were measured in CDCl_3 _(150 MHz); *^b^* Spectra were measured in CD_3_OD (150 MHz).

Compound **2** was obtained as a white, amorphous powder with [α]D^25^ −45.6 (*c* 0.90, CH_3_OH). Its positive ion HRESIMS revealed a pseudo molecular ion peak at *m/z* 515.3711 [M + Na]^+^ (calcd for C_30_H_52_O_5_Na 515.3712), corresponding to the molecular formula C_30_H_52_O_5_, possessing five degrees of unsaturation. IR spectrum showed its absorption at 3234–3587 cm^−1^ for hydroxyl groups, and 1652 cm^−1^ for carbonyl (acetate) group. Comparison of the ^1^H-NMR (Table 1) and ^13^C-NMR (Table 2) data of **2** with those of the known compound **4** [17], revealed that **2** shares the same structure nucleus as **4**, differing from **4** only at the side chain where the C-25 was oxygenated, in agreement with the mass data. The oxygenation of the C-25 caused the ^13^C-NMR resonance of C-26 and C-27 to be shifted significantly downfield (from *δ*_C_ 21.5/21.6 to 26.0/27.2) and two singlet methyl signals appeared in **2** [*δ*_H_ 1.09 (3H, s), 1.10 (3H, s)] instead of two doublet methyls in **4 ** [*δ*_H_ 0.77 (3H, d, *J* = 6.6 Hz), 0.78 (3H, d, *J* = 6.6 Hz)] in the ^1^H-NMR spectrum. According to these data, compound **2** was assigned as the 25-OH derivative of **4**. In addition, the HMBC (Figure 2) correlation from H-6 to ester carbonyl carbon at *δ*_C_ 172.1 (CH_3_*C*O), suggesting that the acetoxy group was positioned at C-6. The assigned relative configuration at C-6 was confirmed by that H-6 (*δ*_H_ 4.68 (1H, t, *J* = 2.4 Hz)) was coupled with H-7α (equatorial) (*δ*_H_ 1.47) with a small coupling constant of 2.4 Hz. Consequently, H-6 was in equatorial orientation, indicating the location of the acetoxy group was at axial position. Furthermore, the NOE cross-peaks observed between H_3_-19 (*δ*_H_1.14) and both H-4β (*δ*_H_ 1.77) and H-2β (*δ*_H_ 2.01), and the absence of NOE correlations between H_3_-19 and both H-3 and H-6 implied that H-3 and H-6 are both α oriented. At the same time the NOE correlations observed between H-4*α* and both H-3 and H-6 further confirmed that H-3 and H-6 were α oriented. The configuration of the side chain of **2** was also confirmed by NOE correlations from H_3_-18 to H-20. So the structure of compound **2** was determined as (24*S*)-ergostane-6-acetate-3β,5α,6β,25-tetraol. 

Compound **3** was obtained as a white, amorphous powder with [α]_D_^25^ −26.6 (*c* 0.50, CH_3_OH). It was found to have the same molecular formula (C_30_H_52_O_5_) as **2**, as determined from high-resolution mass measurements which revealed a pseudo molecular ion peak at *m/z* 515.3710 [M + Na]^+^, (calcd for C_30_H_52_O_5_Na 515.3712). Both compounds (**2** and **3**) showed similarity in the ^1^H NMR and ^13^C NMR spectra (Table 1 and Table 2), with the most significant difference being the chemical shifts of C-5 (*δ*_C_ 75.5, C in **2** *vs. δ*_C_ 30.7, CH in **3**) and C-12 (*δ*_C_ 41.0, CH_2_ in **2** *vs. δ*_C_ 73.7, CH in **3**). This indicated that the location of a hydroxyl group in **3** was different from that of in **2**. The established planar structure of **3** was further supported by the 2D NMR spectra. The diagnostic HMBC correlation from H_3_-18 to C-12 and ^1^H–^1^H COSY correlations between H-12 and H-11 (Figure 2) led the location of the hydroxyl group at C-12. The relative configuration of C-12 was established by comparison with the known compound **8 **(3α-acetoxy-12α-hydroxy-5β-cholan-24-oic acid). The small coupling constant of H-12 (*δ*_H_ 3.99, 1H, t, *J* = 2.6 Hz) in **8 **means that H-12 is at equatorial position according to the literature [23]. Whereas the large coupling constant of H-12 (*δ*_H_ 4.31, 1H, td, *J* = 7.2, 0.6 Hz) in **3** supported the axial position of H-12. Moreover, NOE correlations observed from both H-14 and H-9 to H-12 and the absence of NOE correlations between H_3_-18 and H-12 also implied H-12 was at *α* orientation. The chemical shift of H-3 (*δ*_H_ 4.09) suggested that 3-OH was β oriented and H-5 was *α* oriented by comparison with the ^1^H-NMR data of 3β-hydroxy-5α*-*oxygenated A/B *trans* sterols [24,25]. Based on the above analysis, the relative configuration of **3** was assigned, and the structure was elucidated as (24*S*)-ergostane-6-acetate-3β,6β,12β,25-tetraol.

The structures of compounds **4**, **5** and **6** were identified as 24(*S*)-methylcholestane-3β,5α,6β-triol-6-monoacetate [17], 24-methylenecholestane-3β,5α,6β-triol-6-monoacetate [17], and ergost-24(28)-en-3β,5α,6β-triol [18], respectively, by comparison of their spectroscopic data with those in the literature. 

All the isolated compounds (**1**–**6**) were evaluated for their cytotoxic activity against a panel of five human tumor cell lines (Hela, HL-60, K562, A-549 and SMMC-7721) and lethality toward brine shrimp *A. salina*. Only compound **5** exhibited moderate cytotoxicity against K562 cell line with an IC_50_ value of 3.18 μM. Moreover, compound **5** also displayed strong lethality toward brine shrimp *A. salina* with a LC_50_ value of 0.96 μM. For the other compounds, no cytotoxic activity at the concentration of 10 μM and no lethality toward brine shrimp at 25 μg/mL were found.

## 3. Experimental Section

### 3.1. General Experimental Procedures

Optical rotations were measured in methanol using a JASCO P-1020 digital polarimeter. UV spectra were recorded on a Beckman DU 640 spectrophotometer. IR spectra were measured on a Bruker VECTOR 22 spectrophotometer. ^1^H- and ^13^C-NMR spectra were recorded on a JEOL Eclips-600 spectrometer. ESIMS and HRESIMS were measured on a Q-TOF Ultima Global GAA076 LC mass spectrometer. Silica gel (Qing Dao Hai Yang Chemical Group Co.; 200–300 and 300–400 mesh), octadecylsilyl silica gel (Unicorn; 45–60 μm) and Sephadex LH-20 (Amersham Biosciences) were used for column chromatography (CC). Precoated silica gel plates (Yan Tai Zi Fu Chemical Group Co.; G60, F-254) were used for thin layer chromatography (TLC). Semi-preparative HPLC was performed on a Waters 1525 system using a semi-preparative C18 (Kromasil 7 μm, 10 × 250 mm) column coupled with a Waters 2996 photodiode array detector. 

### 3.2. Animal Materials

Soft coral *Sinularia* sp. was collected from the coral reef of Weizhou Island in the South China Sea in September 2008, and was identified by Prof. Hui Huang, South China Sea Institute of Oceanology, Chinese Academy of Sciences of China. The voucher specimen (No. GX-WZ-2008002-4) was deposited in the Key Laboratory of Marine Drugs, the Ministry of Education, Ocean University of China, Qingdao, China.

### 3.3. Extraction and Isolation

The frozen animals (dry weight 559.7 g) were cut into small pieces and exhaustively extracted with EtOH once (3000 mL) and CHCl_3_–CH_3_OH (1:1) for six times (3000 mL × 6) successively at room temperature. The organic extracts were evaporated to give a residue, which was suspended into H_2_O and partitioned with ethyl acetate. The ethyl acetate fraction was concentrated under reduced pressure to give a residue (28.0 g), which was subjected to gradient silica gel chromatography, eluting with 0%–100% ethyl acetate in light petroleum ether and 20%–100% CH_3_OH in CHCl_3_ to afford nine fractions (Fr. 1–Fr. 9). Fr. 6 was fractionated on silica gel column chromatography eluting with petroleum ether–ethyl acetate (5:1−1:2), and then chromatographed on Sephadex LH-20 eluted with petroleum ether–CHCl_3_–MeOH (2:1:1) to give Fr.61. Further purification of Fr. 61 by semi-preparative HPLC yielded compound **4** (8.4 mg). Fr. 7 and Fr. 8 were firstly isolated by repeated silica gel chromatography, further purified by Sephadex LH-20 (CHCl_3_–MeOH 1:1) and reversed-phase silica gel chromatography, and finally subjected to RP-HPLC to yield **5** (27.8 mg) and **6** (22.9 mg), respectively. Fr. 9 was chromatographed on silica gel eluting with petroleum ether–EtOAc (1:3), and further purified by RP-HPLC (MeOH/H_2_O 90:10, flow rate of 2.0 mL/min) to afford **1** (1.8 mg, *t*_R_ = 26.5 min), **2** (11.3 mg, *t*_R_ = 27.5 min), and **3** (1.3 mg, *t*_R_ = 32.7 min) successively. 

Compound (**1**): White amorphous powder; [α]D^25^ −22.4 (*c* 0.05, CH_3_OH); IR (KBr) ν_max_ 3410–3584 cm^−1^; UV (MeOH) λ_max_: 198 nm, ^1^H NMR (CDCl_3_, 600 MHz) and ^13^C NMR (CDCl_3_, 150 MHz) data in Table 1 and Table 2; HRESIMS *m/z* 455.3498 [M + Na]^+^ (calcd for C_28_H_48_O_3_Na, 455.3496).

Compound (**2**): White amorphous powder; [α]D^25^ −45.6 (*c* 0.90, CH_3_OH); IR (KBr) ν_max_ at 3234–3587, 1652 cm^−1^; UV (MeOH) λ_max_: 195 nm, ^1^H NMR (CD_3_OD, 600 MHz) and ^13^C NMR (CD_3_OD, 150 MHz) data in Table 1 and Table 2; HRESIMS *m/z* 515.3711 [M + Na]^+^ (calcd for C_30_H_52_O_5_Na, 515.3712). 

Compound (**3**): White amorphous powder; [α]D^25^ −26.6 (*c* 0.50, CH_3_OH); IR (KBr) ν_max_ 3434, 3214, 1638 cm^−1^; UV (MeOH) λ_max_: 197 nm, ^1^H NMR (CDCl_3_, 600 MHz) and ^13^C NMR (CDCl_3_, 150 MHz) data in Table 1 and Table 2; HRESIMS *m/z* 515.3710 [M + Na]^+^ (calcd for C_30_H_52_O_5_Na, 515.3712).

### 3.4. Preparation of the (*S*)-and (*R*)-MTPA Esters of **1**

Compound **1** (0.5 mg) was dissolved in 500 μL of pyridine, and dimethylaminopyridine (2.0 mg) and (*R*)-MTPACl (10 μL) were then added in sequence. The reaction mixture was stirred for 24 h at room temperature, and 1 mL of H_2_O was then added. The solution was extracted with 5 mL of CH_2_Cl_2_ and the organic phase was concentrated under reduced pressure. Then the residue was purified by semi-preparative HPLC (100% MeOH) to yield (*S*)-MTPA ester **1s** (0.3 mg, *t*_R_ = 23.40 min). By the same procedure, the (*R*)-MTPA ester **1r **(0.3 mg, *t*_R_ = 26.21 min) was obtained from the reaction of **1** (0.5 mg) with (*S*)-MTPACl (10 μL). Selected ^1^H NMR (CDCl_3_, 600 MHz) of (*S*)-MTPA ester (**1s**): *δ* 7.08–7.43 (5H, Ph), 5.348 (1H, m, H-23), 5.30 (1H, s, H-6), 5.17 (1H, m, H-3), 2.769 (1H, m, H-2a), 2.766 (1H, m, H-4a), 2.046 (1H, m, H-1a), 2.045 (1H, m, H-20), 2.01 (1H, m, H-22), 1.994 (1H, m, H-2b), 1.986 (1H, m, H-4b), 1.350 (1H, m, H-1b), 1.314 (1H, m, H-22), 1.269 (1H, m, H-24), 1.25 (6H, s, H_3_-26 and H_3_-27), 0.994 (3H, d, *J* = 6.6 Hz, H_3_-21), 0.99 (3H, s, H_3_-19), 0.879 (3H, d, *J* = 6.0 Hz, H_3_-28), 0.87 (3H, s, H_3_-18); selected ^1^H NMR (CDCl_3_, 600 MHz) of (*R*)-MTPA ester (**1r**): *δ* 7.08–7.43 (5H, Ph), 5.346 (1H, m, H-23), 5.30 (1H, s, H-6), 5.16 (1H, m, H-3), 2.776 (1H, m, H-2a), 2.764 (1H, m, H-4a), 2.049 (1H, m, H-1a), 2.038 (1H, m, H-20), 2.01 (1H, m, H-22), 1.999 (1H, m, H-2b), 1.984 (1H, m, H-4b), 1.361 (1H, m, H-1b), 1.311 (1H, m, H-22), 1.270 (1H, m, H-24), 1.25 (6H, s, H_3_-26 and H_3_-27), 0.993 (3H, d, *J* = 6.6 Hz, H_3_-21), 0.99 (3H, s, H_3_-19), 0.883 (3H, d, *J* = 6.0 Hz, H_3_-28), 0.87 (3H, s, H_3_-18).

### 3.5. Cytotoxicity Assay

The cytotoxicity against Hela (cervical cancer cells), HL-60 (Human promyelocytic leukemia cells), A-549 (human lung epithelial carcinoma), SMMC-7721 (human hepatocellular carcinoma cell line), and K562 (human immortalised myelogenous leukemia line) cell lines were evaluated by using SRB [26] and MTT [27] methods, respectively, according to the protocols described in previous literature. The test of brine shrimp toxicity on *A. salina* was performed according to standard protocols [28,29]. 

## 4. Conclusions

In our continuing discovery for biological secondary metabolites from marine invertebrates in the South China Sea, this study provided a series of polyoxygenated sterols. The discovery of new compounds **1**–**3** has added to an extremely diverse and complex array of soft coral sterols. Further studies should be conducted to unambiguously establish their absolute configurations by total synthesis as well as to understand their biological/ecological roles in the life cycle of the animal.

## Supplementary Files

Supplementary File 1: PDF-Document (PDF, 510 KB)
