# Supplementary material for: Polyoxygenated Sterols from the South China Sea Soft Coral Sinularia sp
Source: Mar Drugs. 2012 Jun 26;10(7):1422–32. doi: 10.3390/md10071422 (PMC3407921; doi:10.3390/md10071422)

Supplementary Materials

Figure S1. HR-ESI-MS of compound **1**.
Figure S2. ESI-MS of compound **1**.
Figure S3. ^{1}H NMR spectrum ($CDCl_3$, 600 MHz) of compound **1**.
Figure S4. ^{13}C NMR and DEPT ($CDCl_3$, 125 MHz) spectra of compound **1**.
Figure S5. HMQC spectrum of compound **1**.
Figure S6. ^{1}H-^{1}H COSY spectrum of compound **1**.
Figure S7. HMBC spectrum of compound **1**.
Figure S8. NOESY spectrum of compound **1**.
Figure S9. HR-ESI-MS of compound **2**.
Figure S10. ESI-MS of compound **2**.
Figure S11. ^{1}H NMR spectrum (CD_3OD, 600 MHz) of compound **2**.
Figure S12. ^{13}C NMR and DEPT (CD_3OD, 125 MHz) spectra of compound **2**.
Figure S13. HMQC spectrum of compound **2**.
Figure S14. ^{1}H-^{1}H COSY spectrum of compound **2**.
Figure S15. HMBC spectrum of compound **2**.
Figure S16. NOESY spectrum of compound **2**.
Figure S17. HR-ESI-MS of compound **3**.
Figure S18. ESI-MS of compound **3**.
Figure S19. ^{1}H NMR spectrum ($CDCl_3$, 600 MHz) of compound **3**.
Figure S20. ^{13}C NMR and DEPT ($CDCl_3$, 125 MHz) spectra of compound **3**.
Figure S21. HMQC spectrum of compound **3**.
Figure S22. ^{1}H-^{1}H COSY spectrum of compound **3**.
Figure S23. HMBC spectrum of compound **3**.
Figure S24. NOESY spectrum of compound **3**.
Figure S25. ^{1}H NMR spectrum ($CDCl_3$, 600 MHz) of **1s**.
Figure S26. ^{1}H-^{1}H COSY spectrum of **1s**.
Figure S27. ^{1}H NMR spectrum ($CDCl_3$, 600 MHz) of **1r**.
Figure S28. ^{1}H-^{1}H COSY spectrum of **1r**.

Figure S1. HR-ESI-MS of compound **1**.

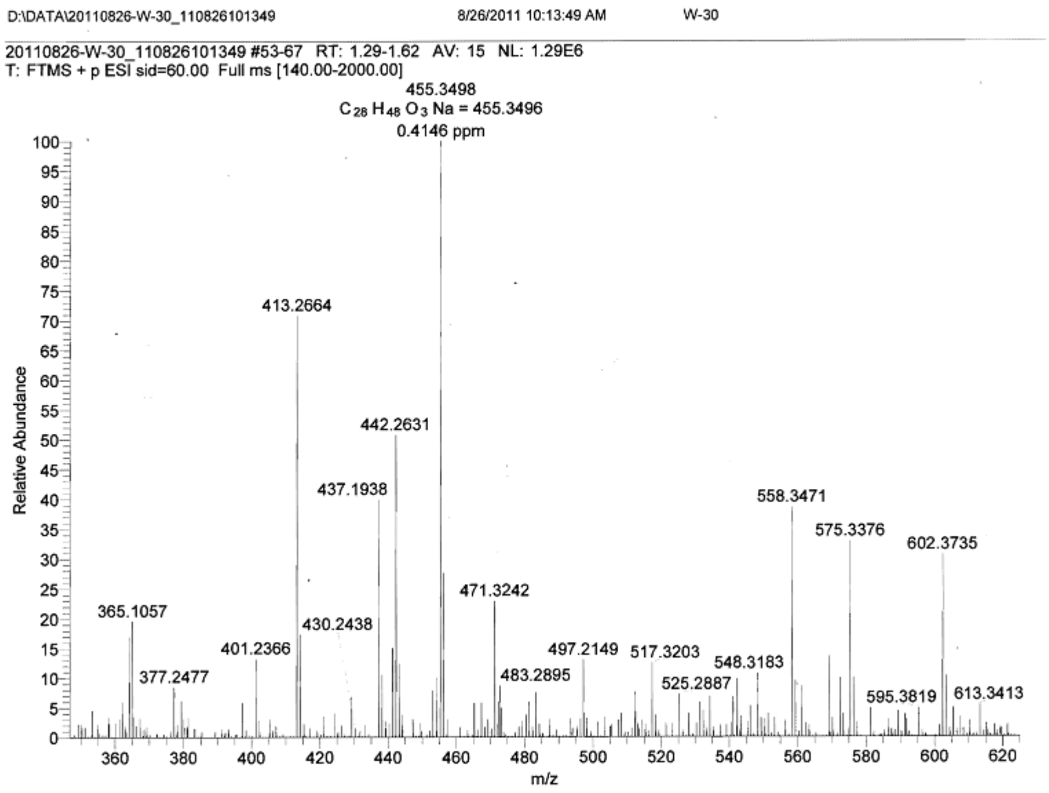

Figure S2. ESI-MS of compound **1**.

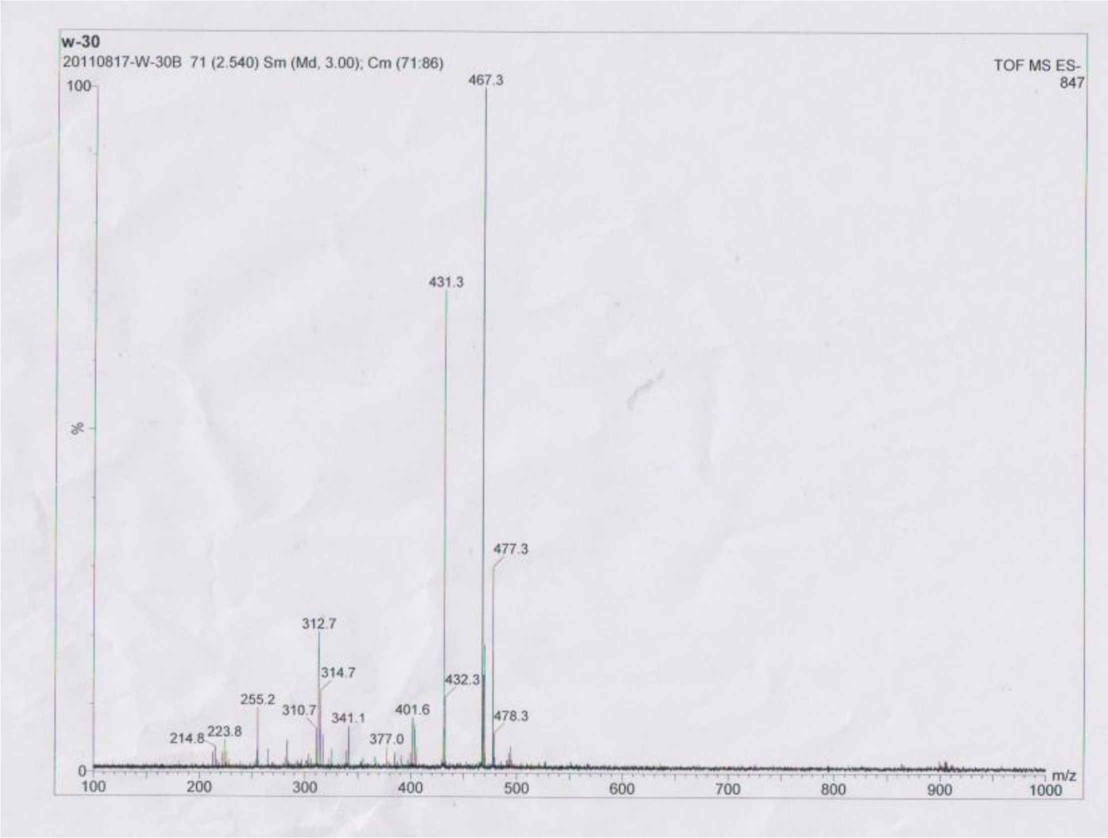

Figure S3. ^{1}H NMR spectrum ($CDCl_3$, 600 MHz) of compound **1**.

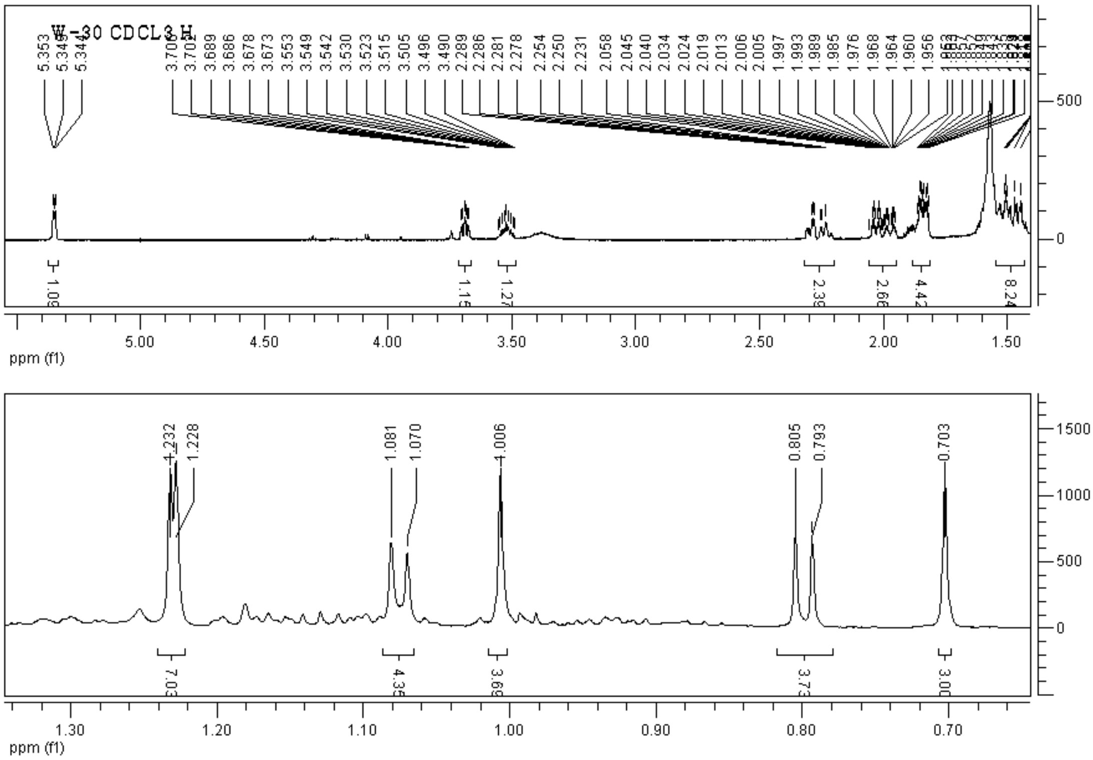

Figure S4. ^{13}C NMR and DEPT ($CDCl_3$, 125 MHz) spectra of compound **1**.

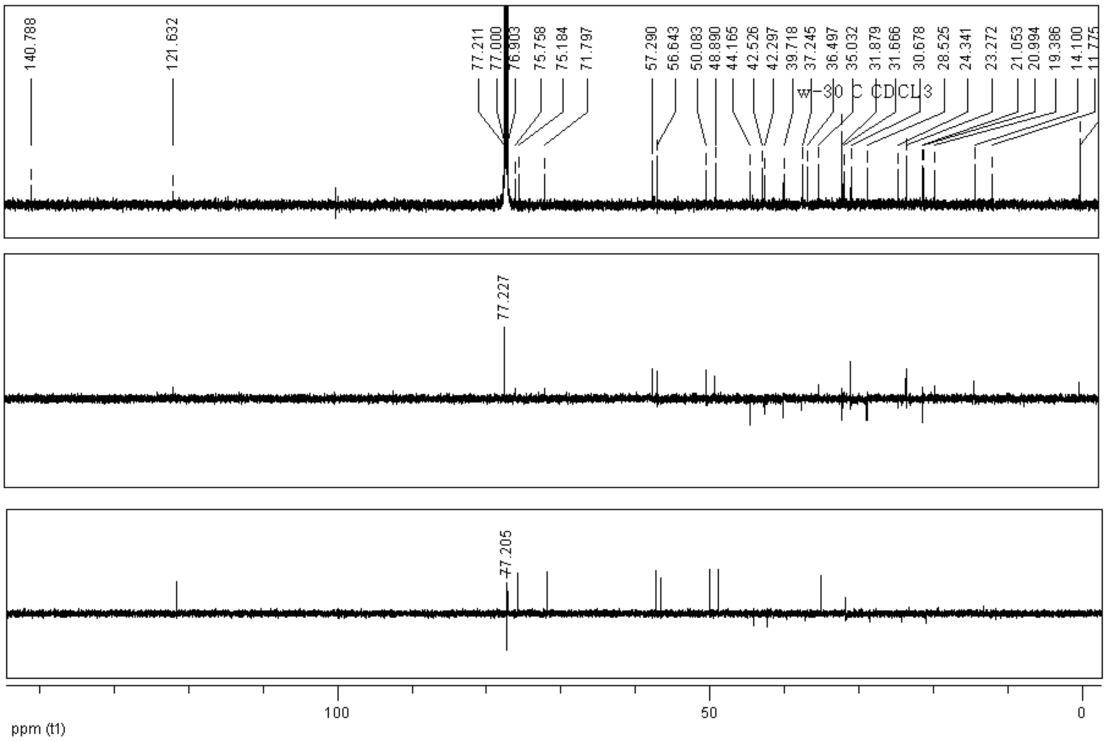

Figure S5. HMQC spectrum of compound **1**.

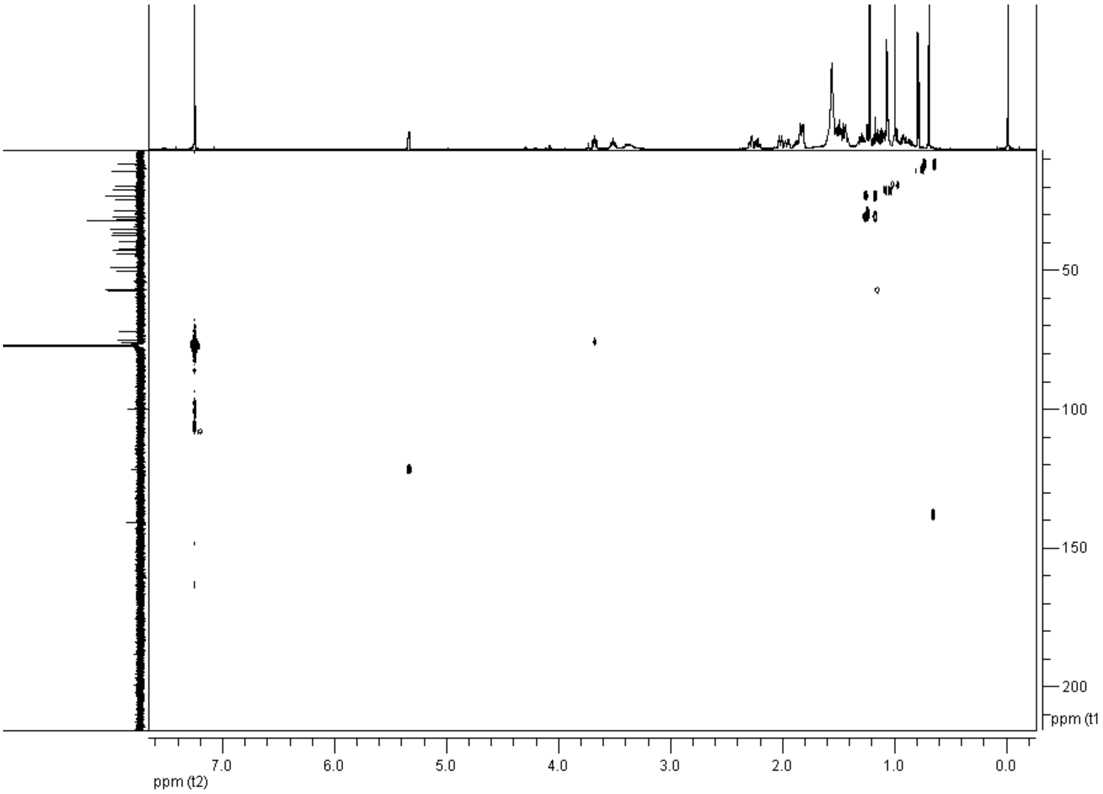

Figure S6. ^{1}H-^{1}H COSY spectrum of compound **1**.

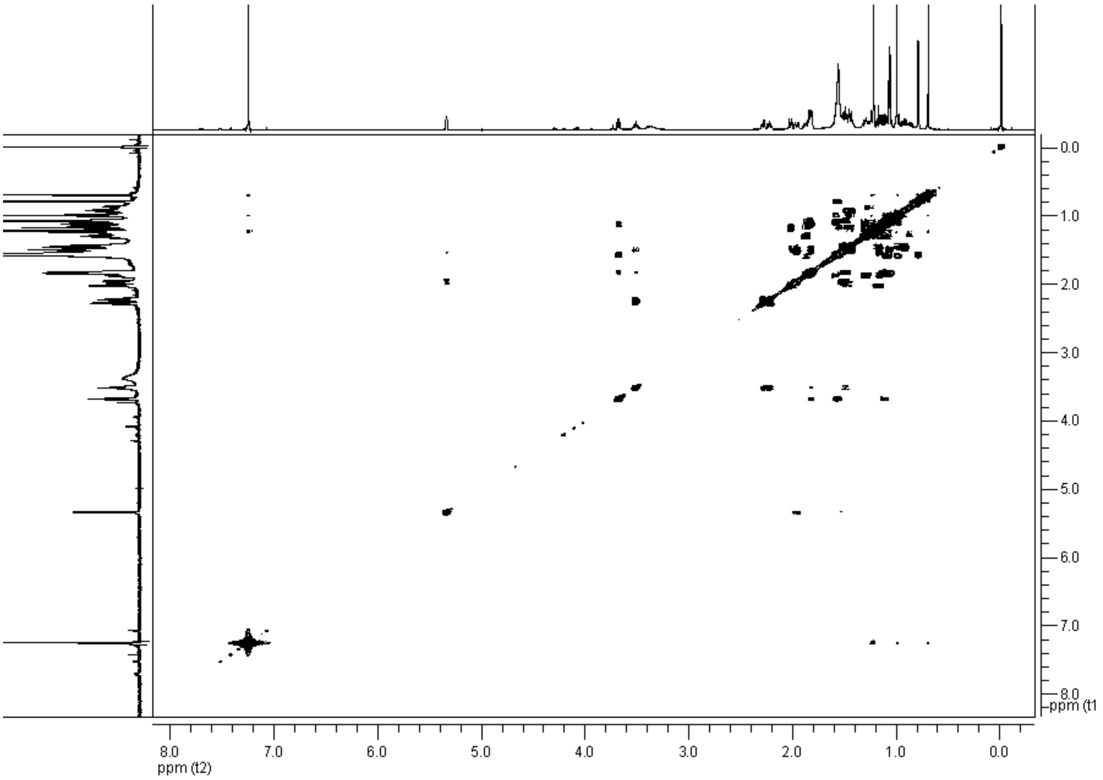

Figure S7. HMBC spectrum of compound **1**.

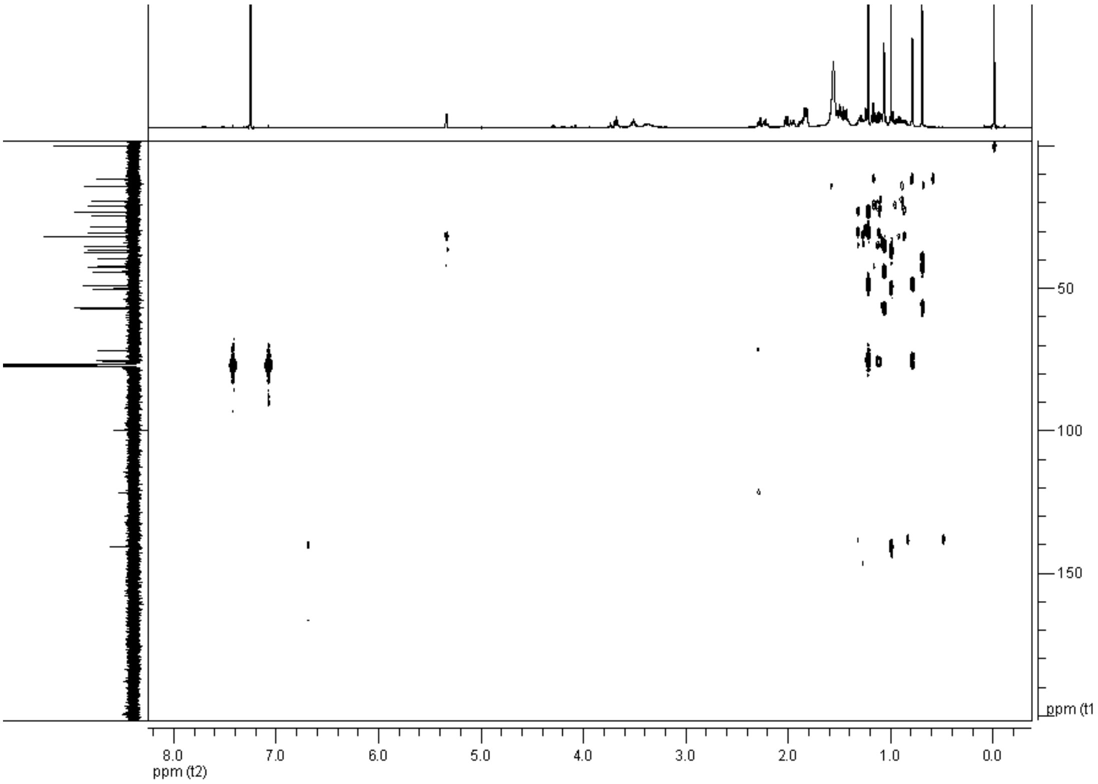

Figure S8. NOESY spectrum of compound **1**.

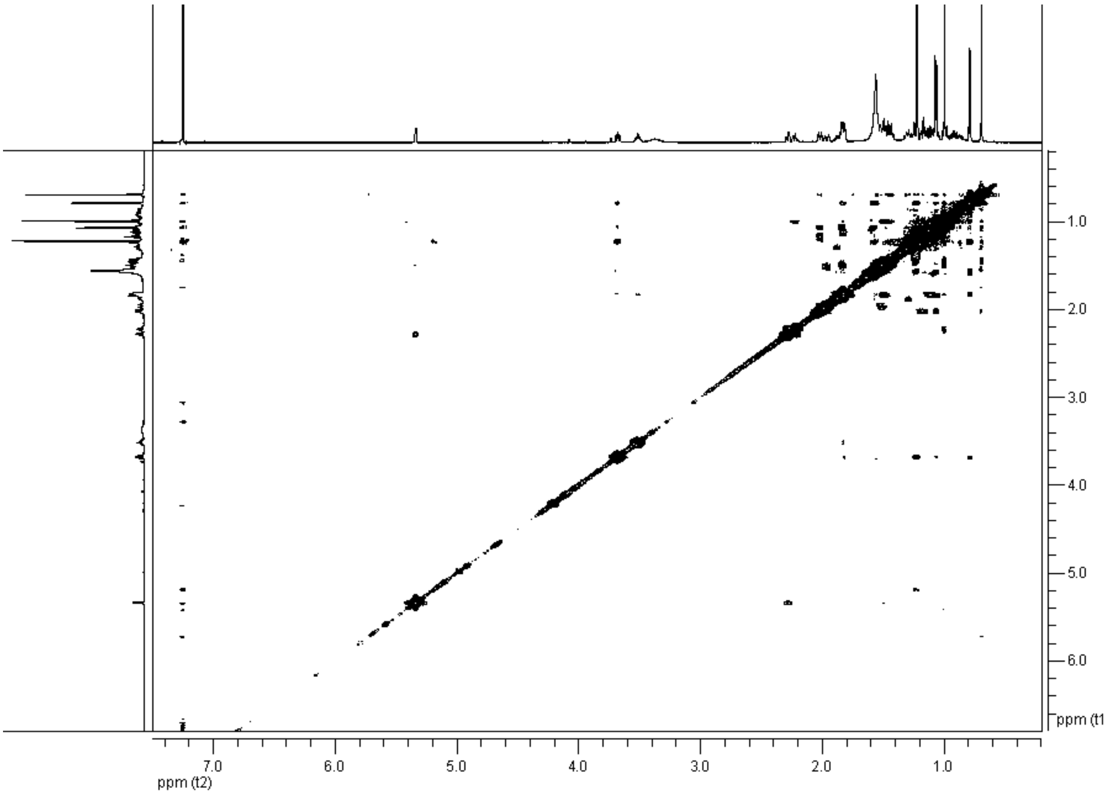

Figure S9. HR-ESI-MS of compound **2**.

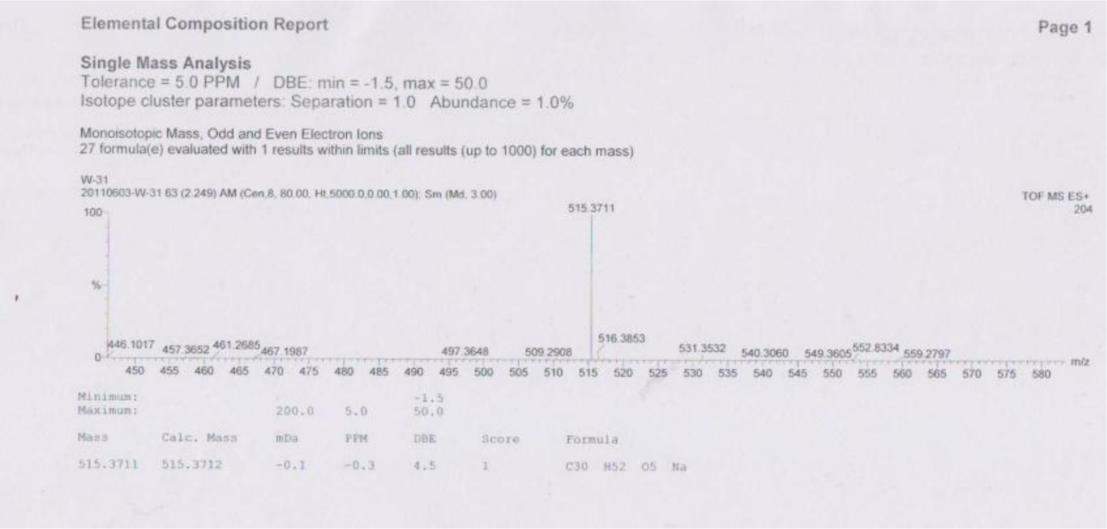
Elemental Composition Report Page 1

Single Mass Analysis
Tolerance = 5.0 PPM / DBE: min = -1.5, max = 50.0
Isotope cluster parameters: Separation = 1.0 Abundance = 1.0%

Monoisotopic Mass, Odd and Even Electron Ions
27 formula(e) evaluated with 1 results within limits (all results (up to 1000) for each mass)

Minimum:				-1.5		
Maximum:		200.0	5.0	50.0		
Mass	Calc. Mass	mDa	PPM	DBE	Score	Formula
515.3711	515.3712	-0.1	-0.3	4.5	1	C30 H52 O5 Na

Figure S10. ESI-MS of compound **2**.

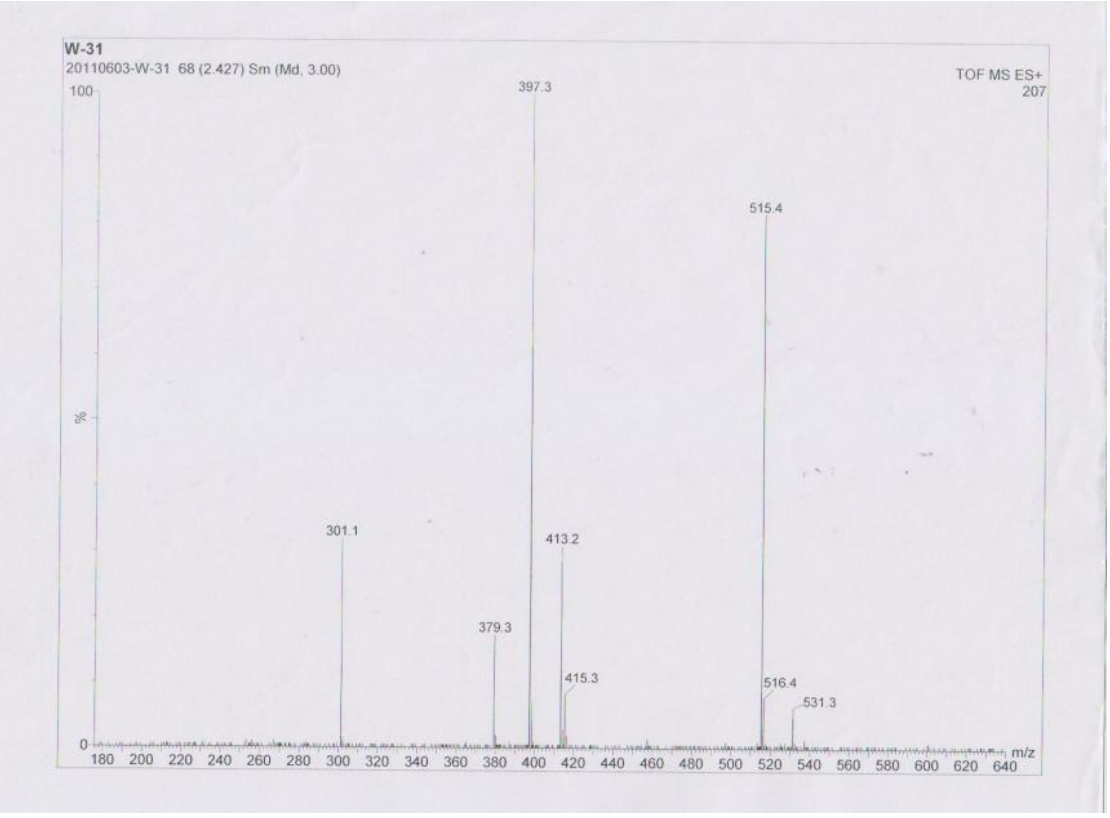

Figure S11. ^{1}H NMR spectrum (CD_3OD, 600 MHz) of compound **2**.

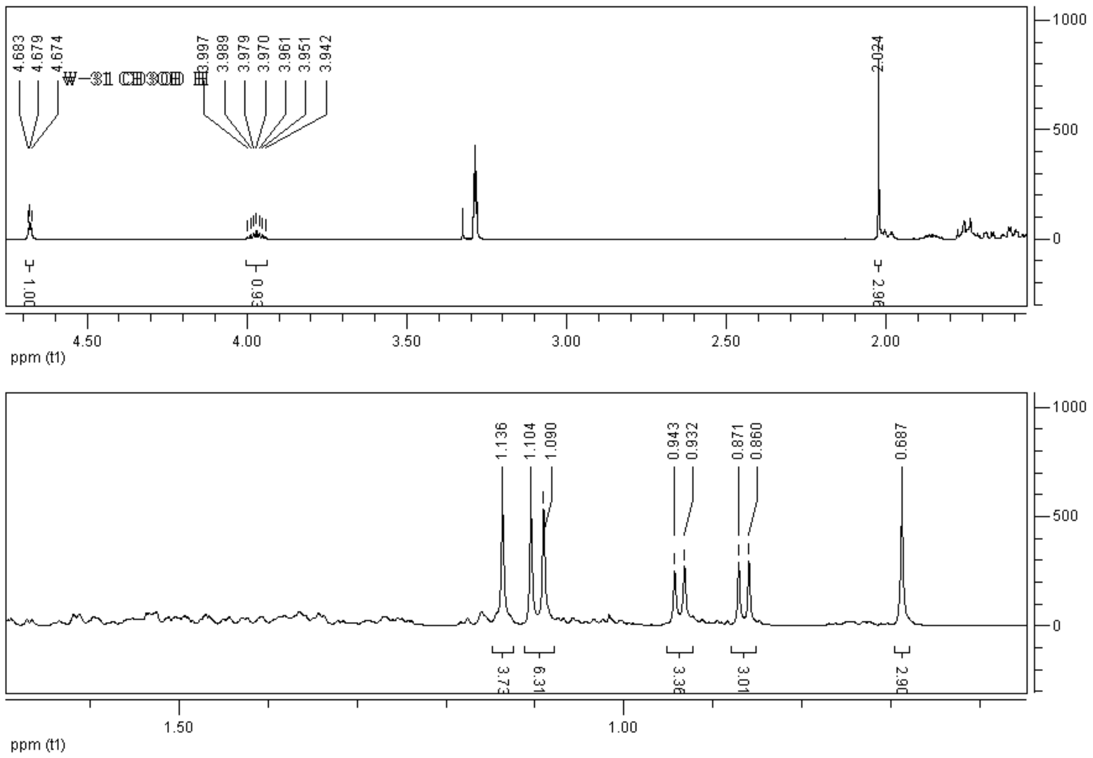

Figure S12. ^{13}C NMR and DEPT (CD_3OD, 125 MHz) spectra of compound **2**.

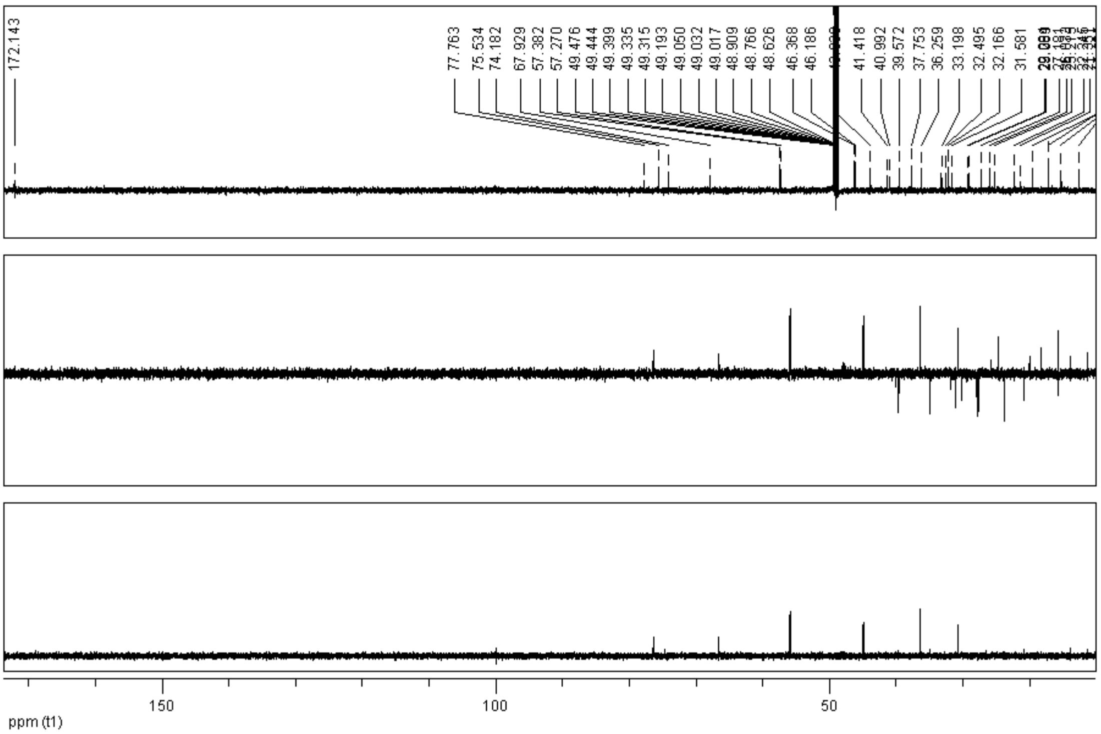

Figure S13. HMQC spectrum of compound **2**.

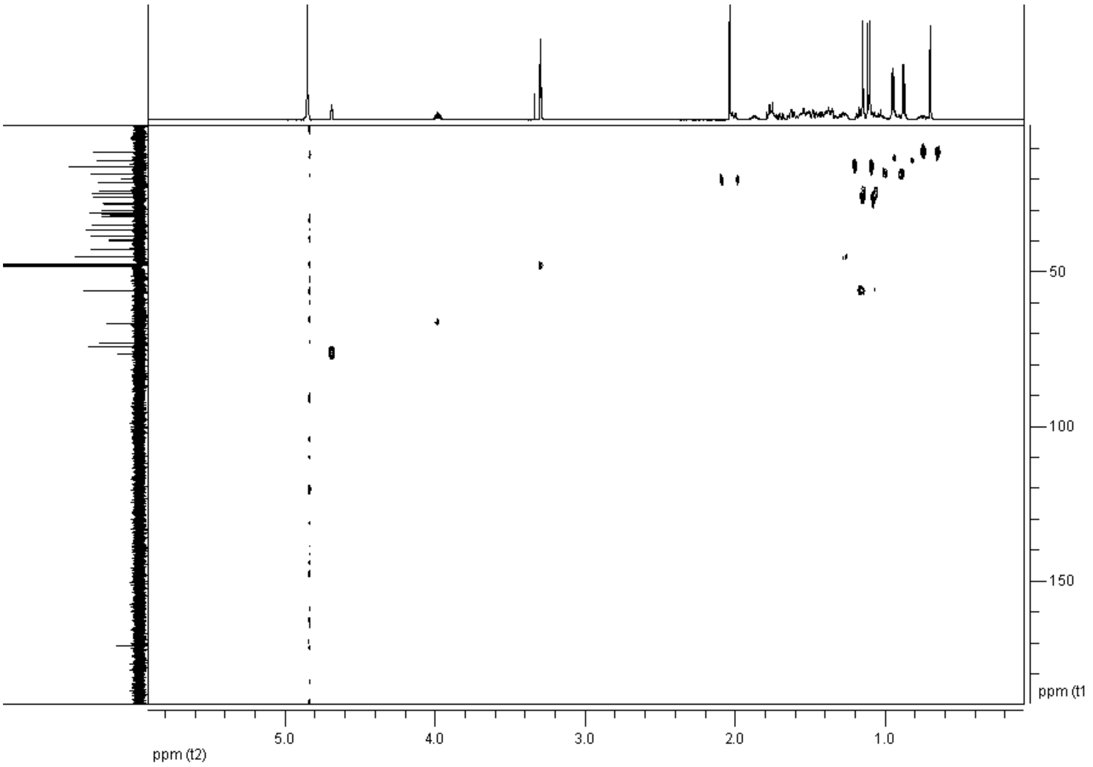

Figure S14. ^{1}H-^{1}H COSY spectrum of compound **2**.

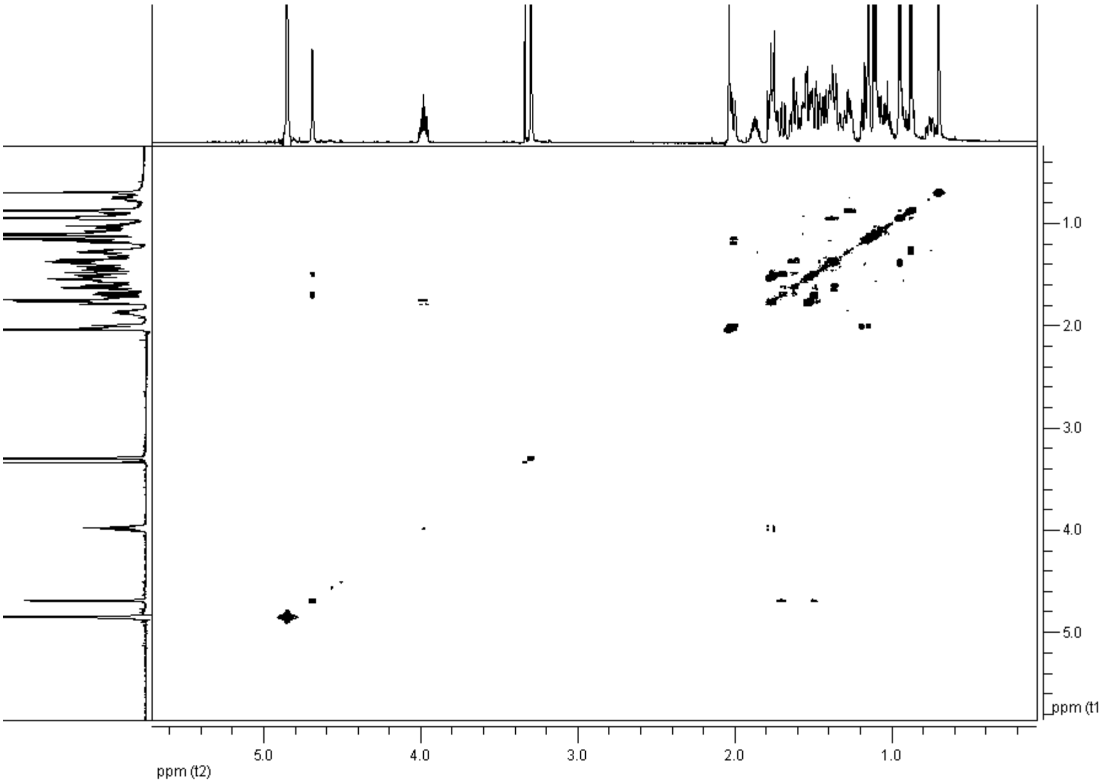

Figure S15. HMBC spectrum of compound **2**.

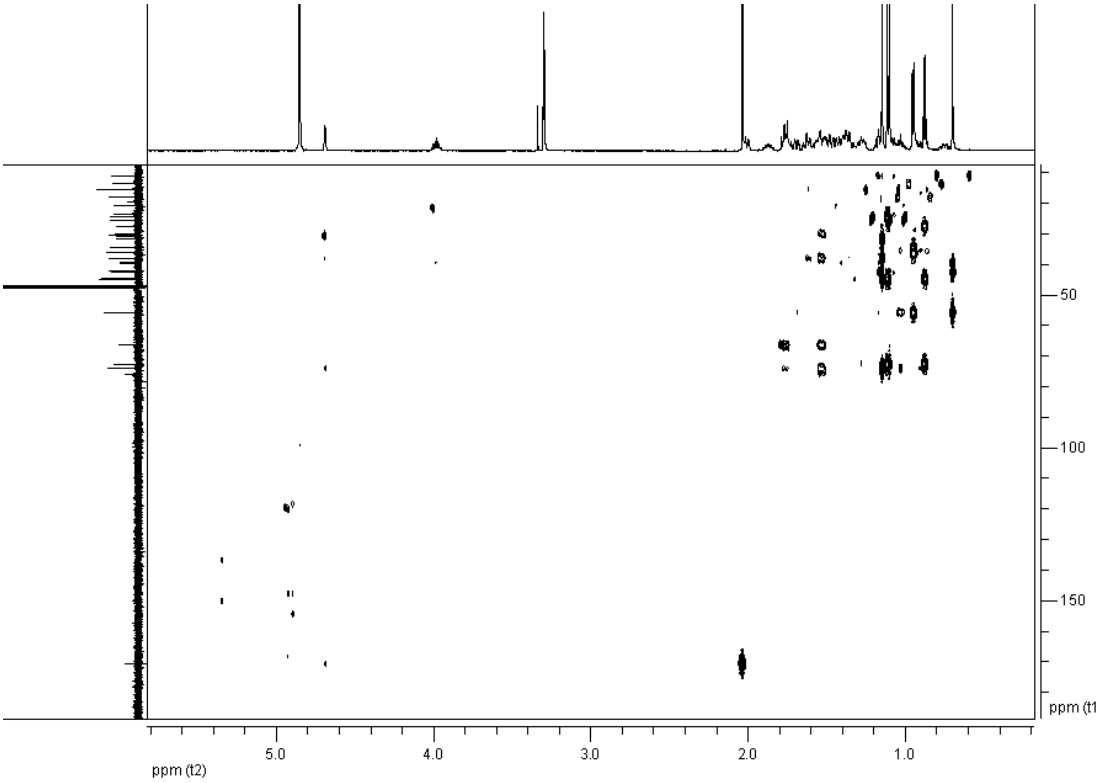

Figure S16. NOESY spectrum of compound **2**.

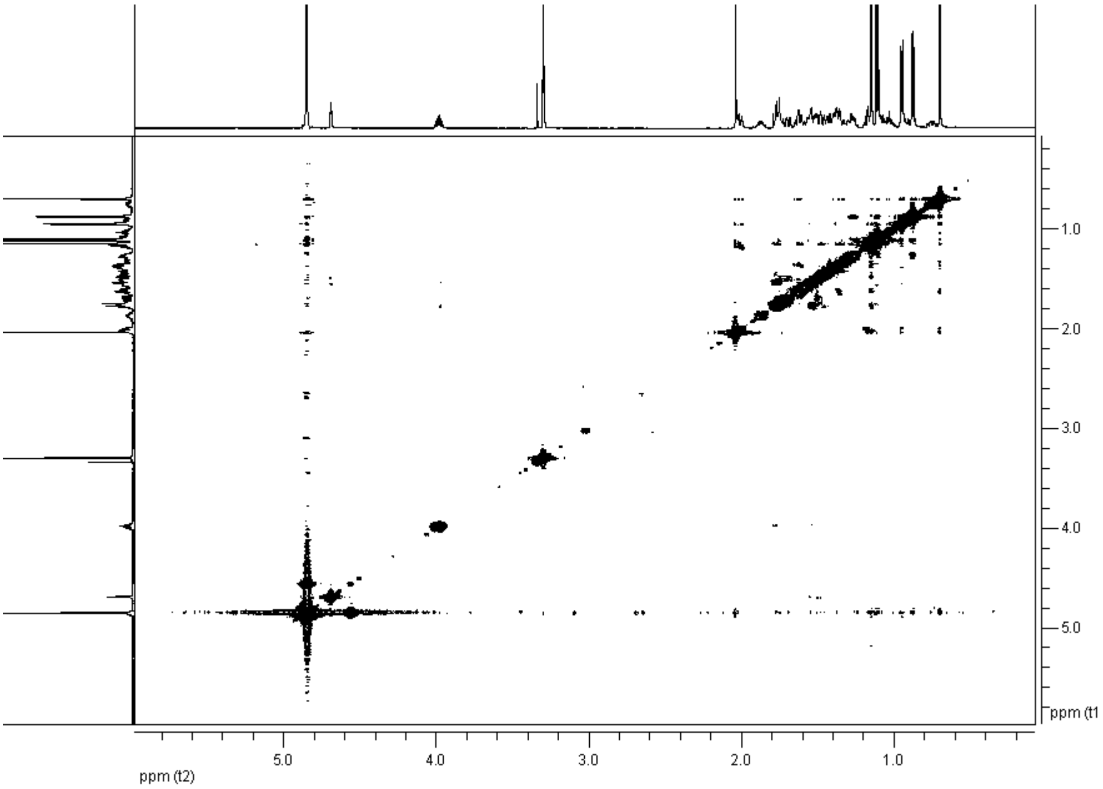

Figure S17. HR-ESI-MS of compound **3**.

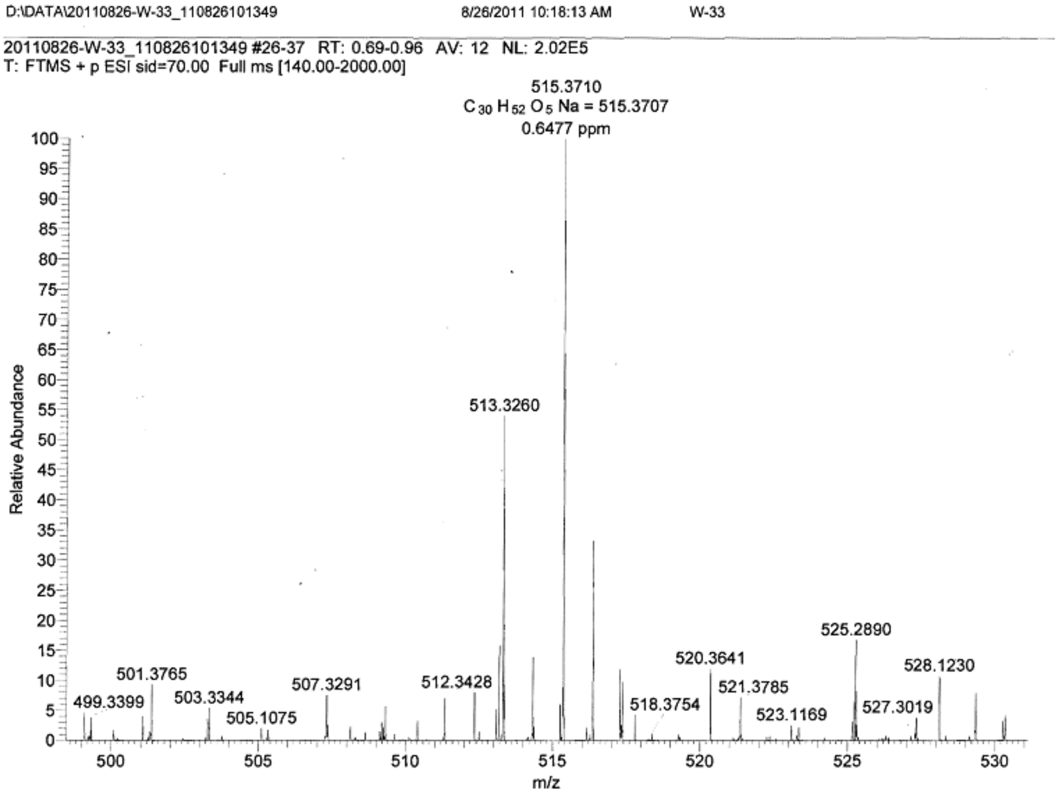

Figure S18. ESI-MS of compound **3**.

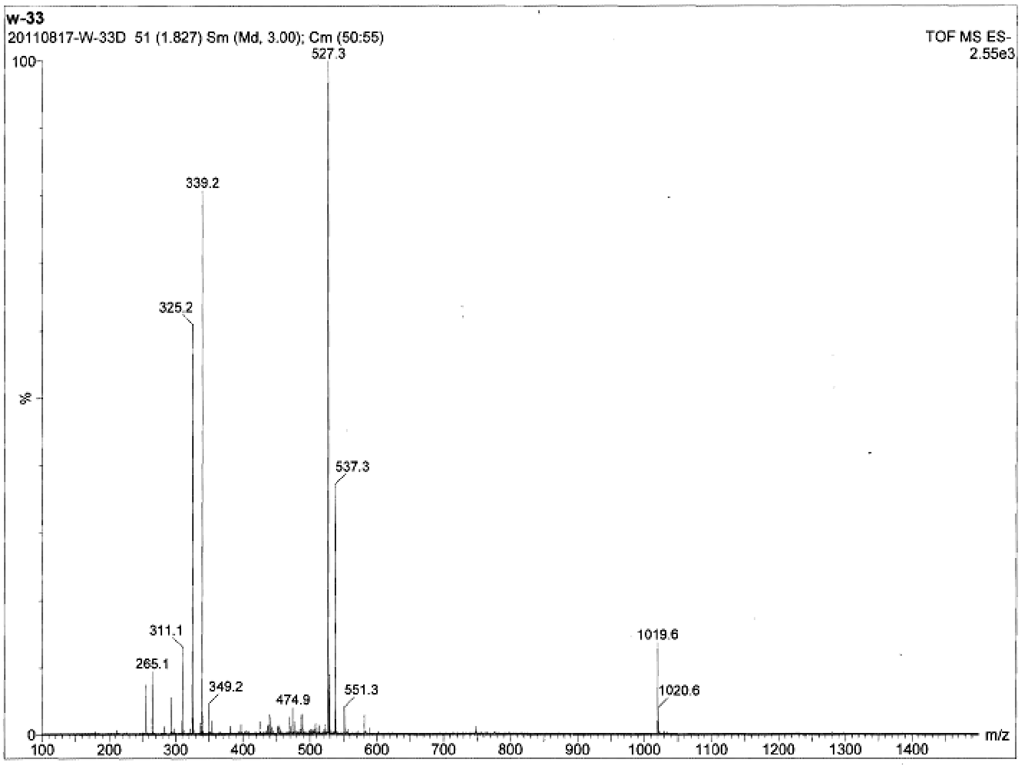

Figure S19. ^{1}H NMR spectrum ($CDCl_3$, 600 MHz) of compound **3**.

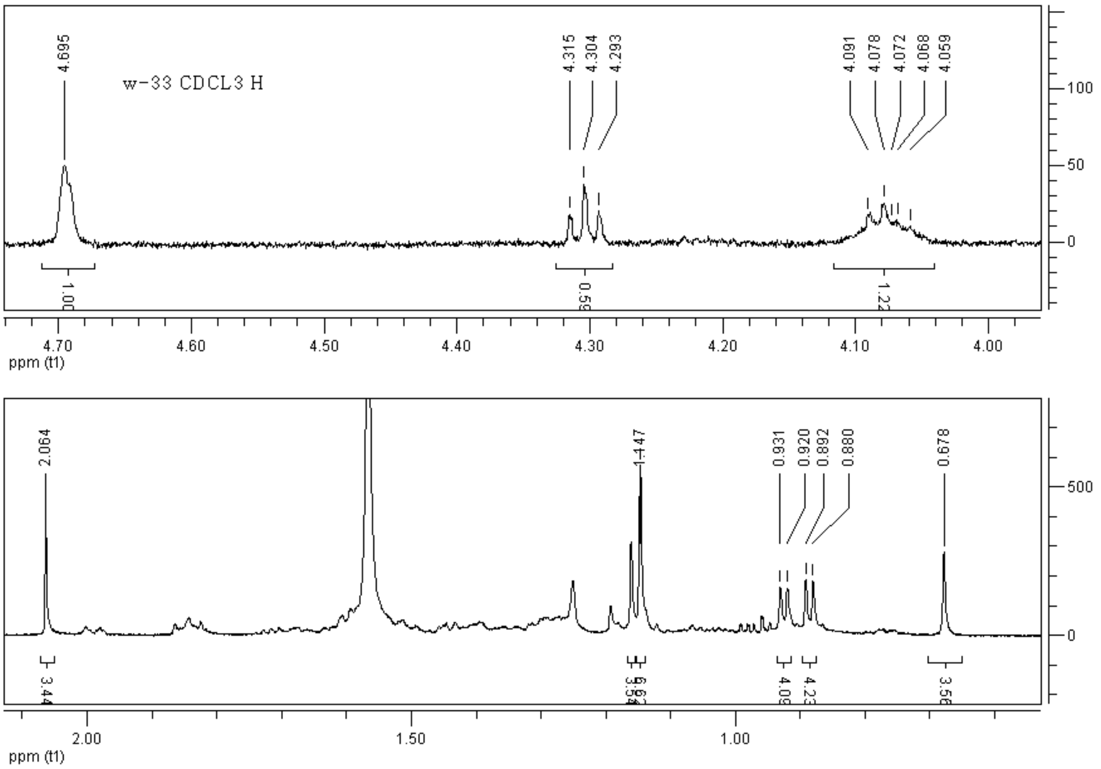

Figure S20. ^{13}C NMR spectrum ($CDCl_3$, 125 MHz) of compound **3**.

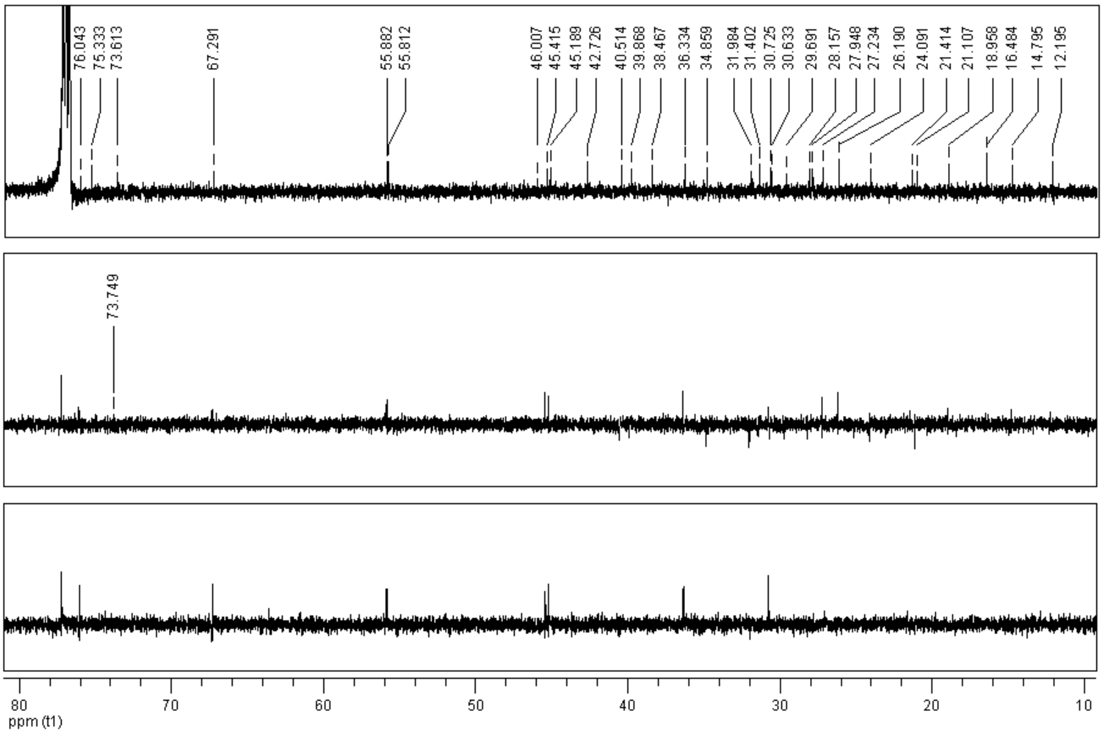

Figure S21. HMQC spectrum of compound **3**.

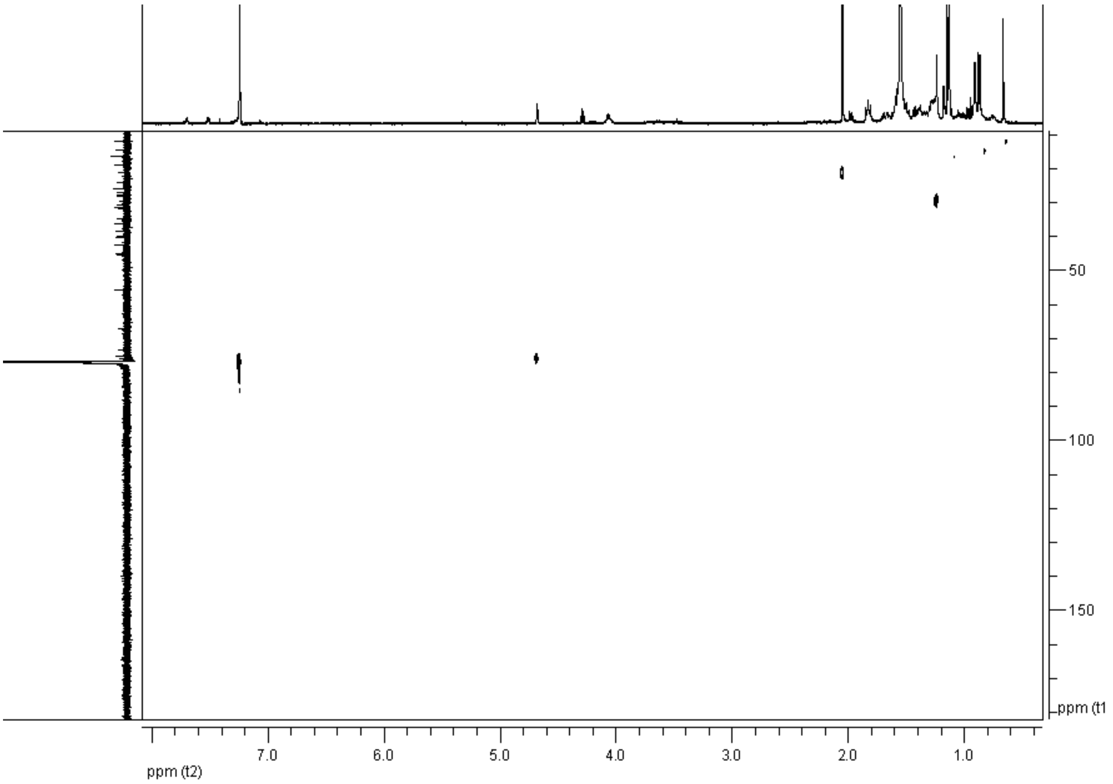

Figure S22. ^{1}H-^{1}H COSY spectrum of compound **3**.

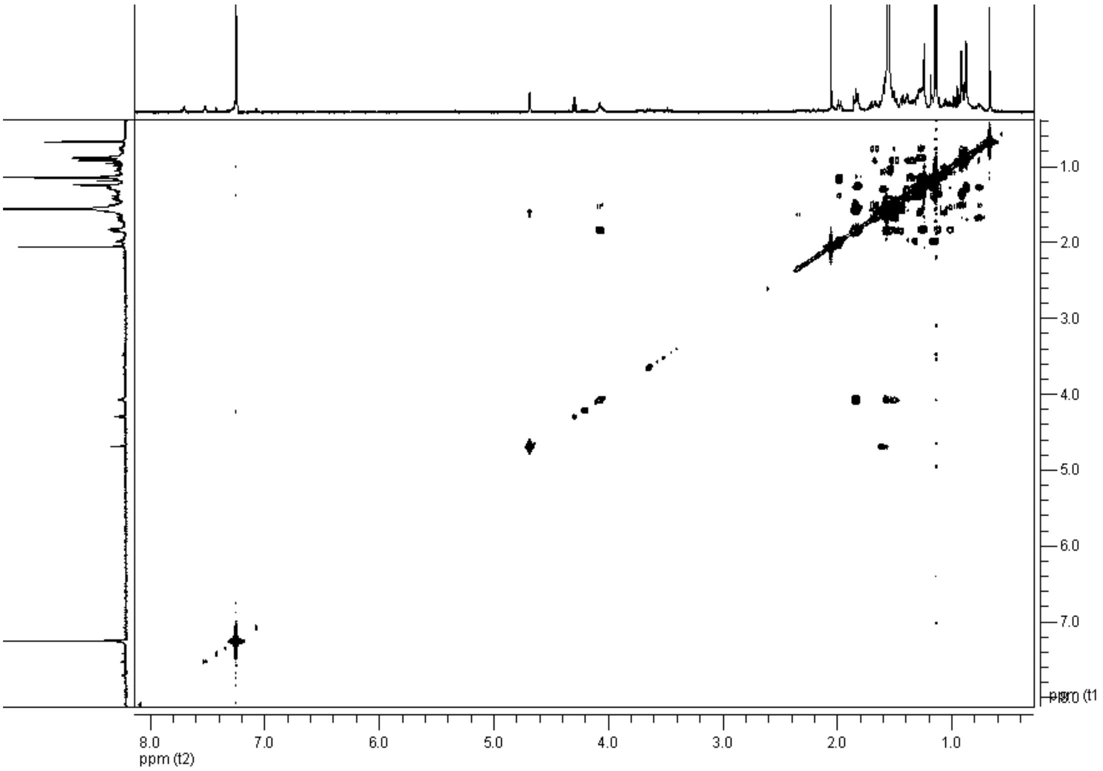

Figure S23. HMBC spectrum of compound **3**.

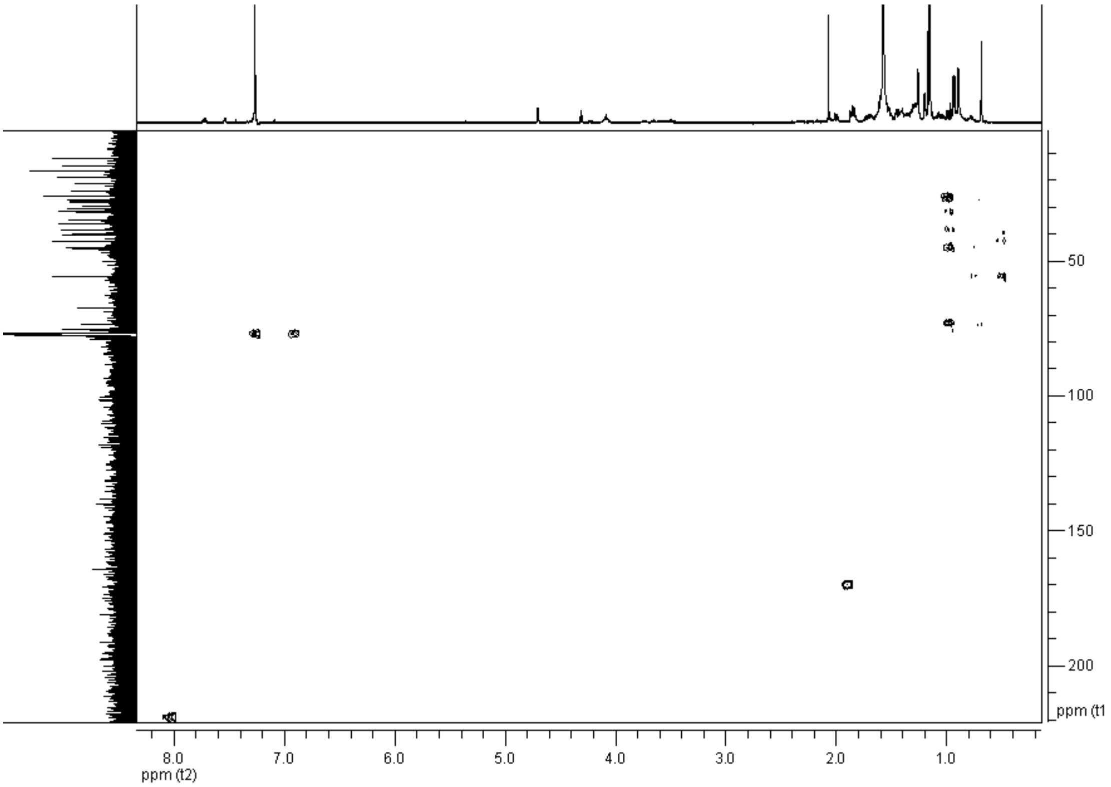

Figure S24. NOESY spectrum of compound **3**.

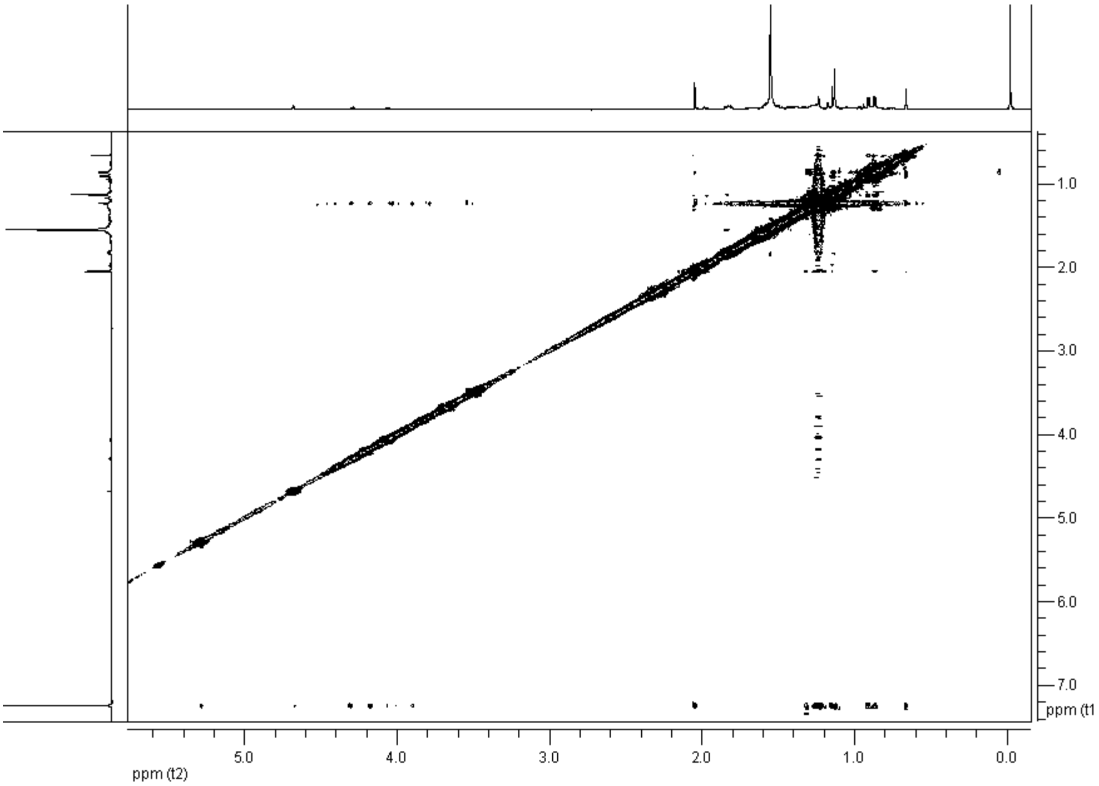

Figure S25. ^{1}H NMR spectrum ($CDCl_3$, 600 MHz) of **1s**.

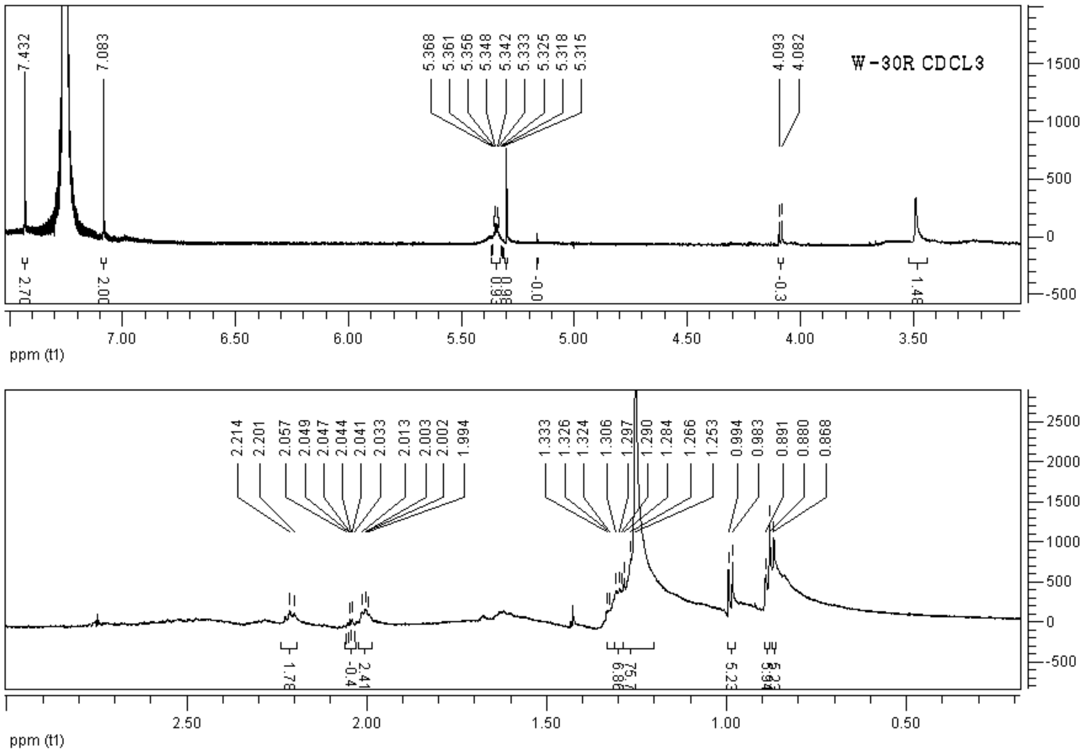

Figure S26. ^{1}H-^{1}H COSY spectrum of **1s**.

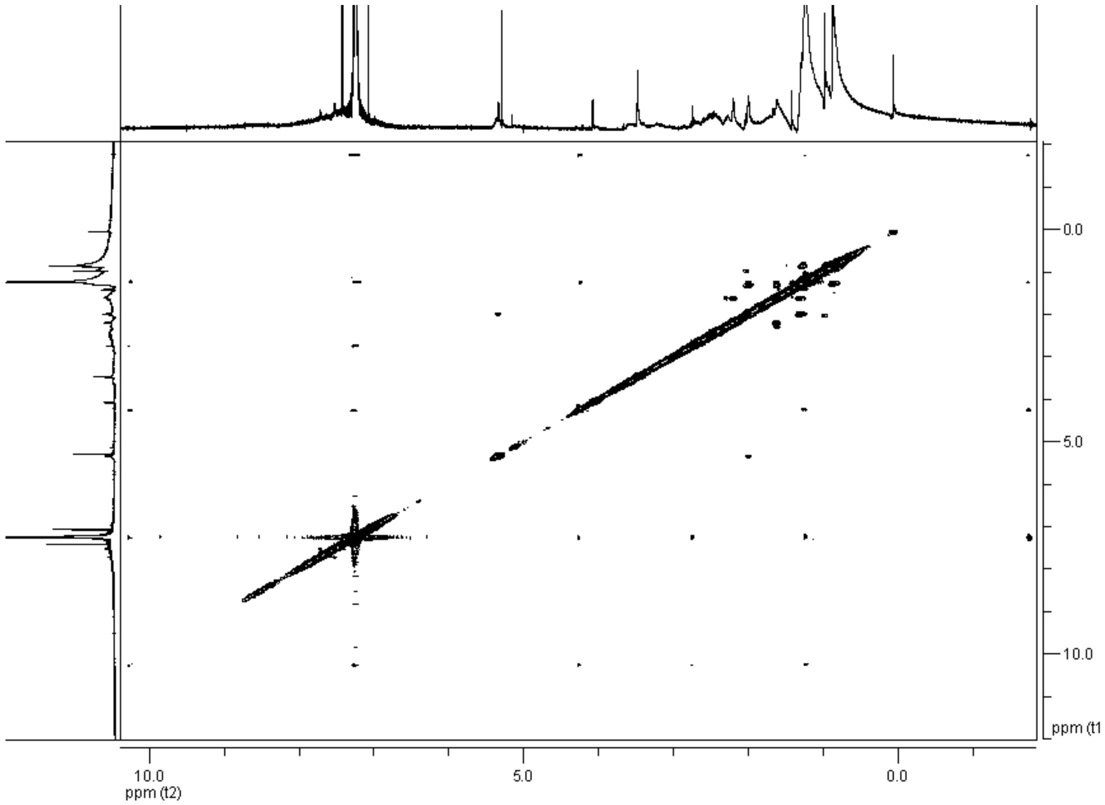

Figure S27. ^{1}H NMR spectrum ($CDCl_3$, 600 MHz) of **1r**.

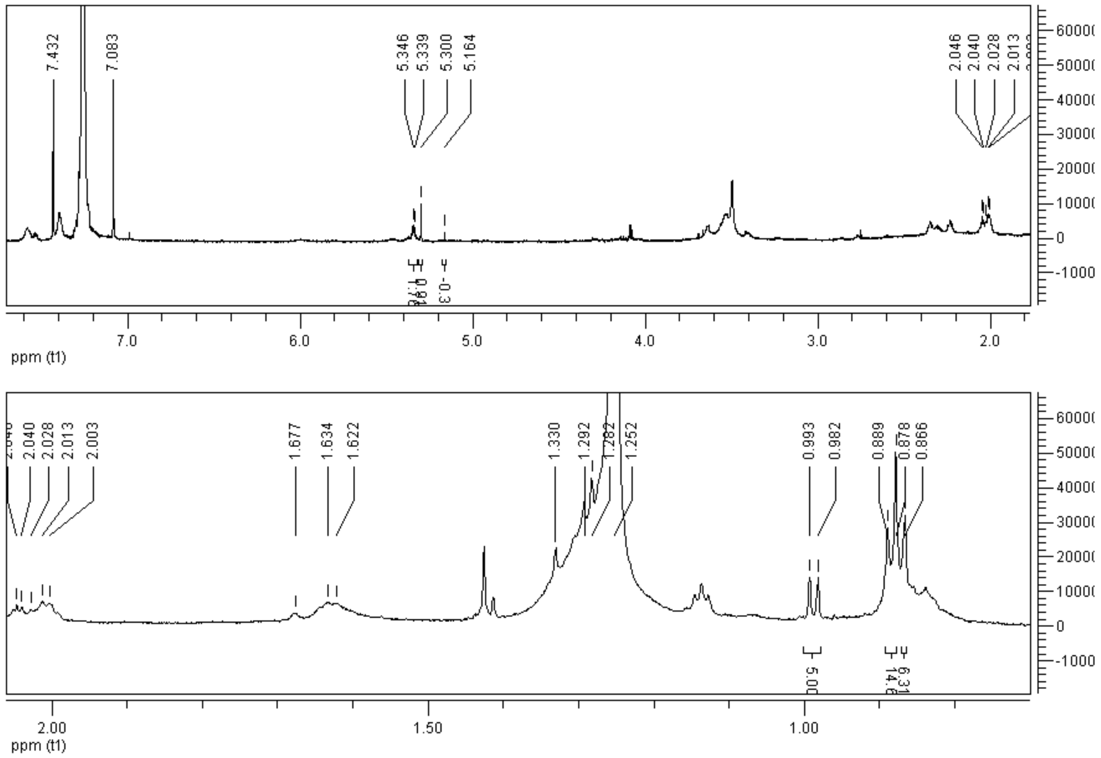

Figure S28. ^{1}H-^{1}H COSY spectrum of **1r**.

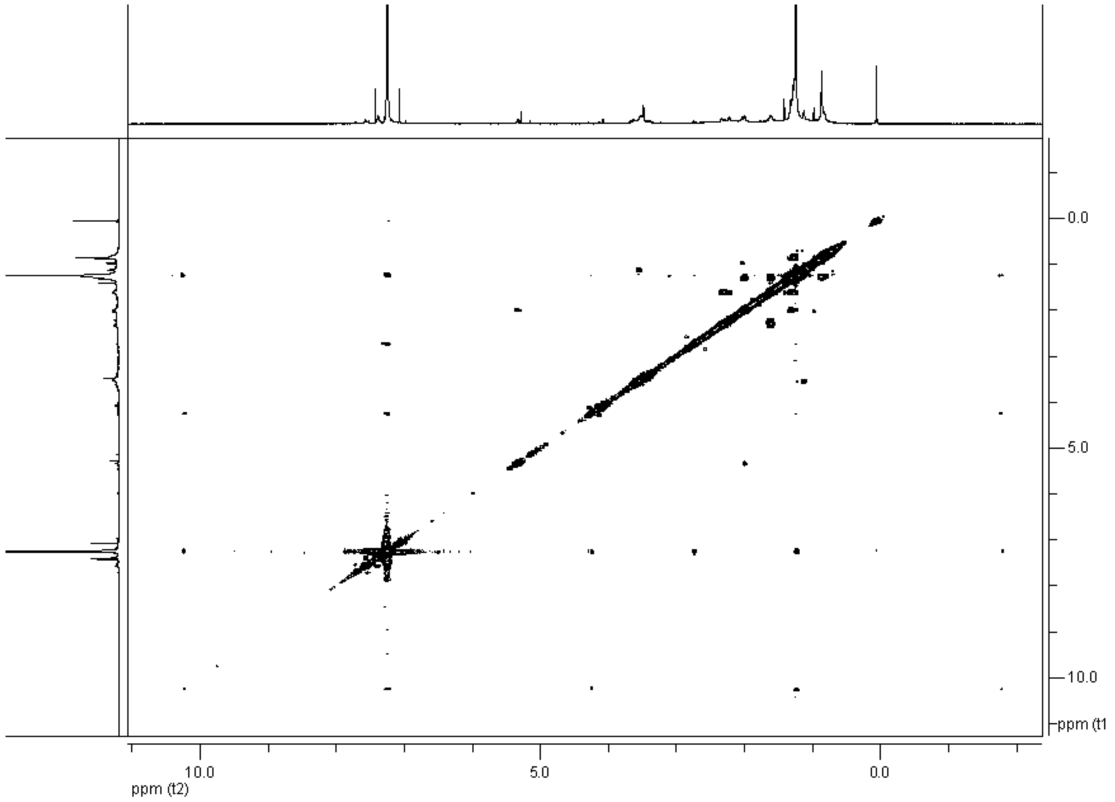

Supplement: Supplementary File 1: — PDF-Document (PDF, 510 KB) [file marinedrugs-10-01422-s001.pdf]
